# Double-Edge Sword of Sustained ROCK Activation in Prion Diseases through Neuritogenesis Defects and Prion Accumulation

**DOI:** 10.1371/journal.ppat.1005073

**Published:** 2015-08-04

**Authors:** Aurélie Alleaume-Butaux, Simon Nicot, Mathéa Pietri, Anne Baudry, Caroline Dakowski, Philippe Tixador, Hector Ardila-Osorio, Anne-Marie Haeberlé, Yannick Bailly, Jean-Michel Peyrin, Jean-Marie Launay, Odile Kellermann, Benoit Schneider

**Affiliations:** 1 INSERM, UMR-S 1124, Paris, France; 2 Université Paris Descartes, Sorbonne Paris Cité, UMR-S 1124, Paris, France; 3 CNRS UMR 8256, Biological Adaptation and Ageing, Paris, France; 4 Sorbonne Université, UPMC Université, Paris 06, UMR 8256, B2A, Biological Adaptation and Ageing, Institut de Biologie Paris Seine, Paris, France; 5 Cytologie et Cytopathologie Neuronales, Institut des Neurosciences Cellulaires et Intégratives, CNRS UPR 3212, Strasbourg, France; 6 AP-HP, Service de Biochimie, INSERM UMR-S942, Hôpital Lariboisière, Paris, France; 7 Pharma Research Department, Hoffmann La Roche Ltd, Basel, Switzerland; Georg-August University Goettingen, GERMANY

## Abstract

In prion diseases, synapse dysfunction, axon retraction and loss of neuronal polarity precede neuronal death. The mechanisms driving such polarization defects, however, remain unclear. Here, we examined the contribution of RhoA-associated coiled-coil containing kinases (ROCK), key players in neuritogenesis, to prion diseases. We found that overactivation of ROCK signaling occurred in neuronal stem cells infected by pathogenic prions (PrP^Sc^) and impaired the sprouting of neurites. In reconstructed networks of mature neurons, PrP^Sc^-induced ROCK overactivation provoked synapse disconnection and dendrite/axon degeneration. This overactivation of ROCK also disturbed overall neurotransmitter-associated functions. Importantly, we demonstrated that beyond its impact on neuronal polarity ROCK overactivity favored the production of PrP^Sc^ through a ROCK-dependent control of 3-phosphoinositide-dependent kinase 1 (PDK1) activity. In non-infectious conditions, ROCK and PDK1 associated within a complex and ROCK phosphorylated PDK1, conferring basal activity to PDK1. In prion-infected neurons, exacerbated ROCK activity increased the pool of PDK1 molecules physically interacting with and phosphorylated by ROCK. ROCK-induced PDK1 overstimulation then canceled the neuroprotective α-cleavage of normal cellular prion protein PrP^C^ by TACE α-secretase, which physiologically precludes PrP^Sc^ production. In prion-infected cells, inhibition of ROCK rescued neurite sprouting, preserved neuronal architecture, restored neuronal functions and reduced the amount of PrP^Sc^. In mice challenged with prions, inhibition of ROCK also lowered brain PrP^Sc^ accumulation, reduced motor impairment and extended survival. We conclude that ROCK overactivation exerts a double detrimental effect in prion diseases by altering neuronal polarity and triggering PrP^Sc^ accumulation. Eventually ROCK emerges as therapeutic target to combat prion diseases.

## Author Summary

Transmissible Spongiform Encephalopathies (TSEs), commonly named prion diseases, are caused by deposition in the brain of pathogenic prions PrP^Sc^ that trigger massive neuronal death. Because of our poor understanding of the mechanisms sustaining prion-induced neurodegeneration, there is to date no effective medicine to combat TSEs. The current study demonstrates that ROCK kinases are overactivated in prion-infected cells and contribute to prion pathogenesis at two levels. First, PrP^Sc^-induced ROCK overactivation affects neuronal polarity with synapse disconnection, axon/dendrite degradation, and disturbs neuronal functions. Second, ROCK overactivity amplifies the production of pathogenic prions. The pharmacological inhibition of ROCK protects diseased neurons from PrP^Sc^ toxicity by preserving neuronal architecture and functions and lowering PrP^Sc^ level. Inhibition of ROCK in prion-infected mice reduces brain PrP^Sc^ levels, improves motor activity and extends lifespan. This study opens up new avenues to design ROCK-based therapeutic strategies to fight TSEs.

## Introduction

In neurodegenerative disorders including Transmissible Spongiform Encephalopathies (TSEs), it is now admitted that neuronal death is a late event in the neurodegenerative process preceded by an early loss of neuronal polarity at the root of behavioral and cognitive deficits [1–4]. In TSEs, synapse retraction and progressive axonal degeneration correlate with brain accumulation of the scrapie protein (PrP^Sc^), which is the essential component of infectious prions [5]. PrP^Sc^ is an abnormally folded self-propagating isoform of cellular prion protein (PrP^C^), a physiological cell-surface glycosylphosphatidylinositol(GPI)-anchored protein. The neurotoxic effects of PrP^Sc^ depend on the neuronal expression of PrP^C^ since the suppression of PrP^C^ in neurons of infected mice, just prior to the clinical phase, hampers PrP^Sc^-induced neuronal loss [6–8]. For instance, prion-associated neurotoxicity relates to subversion of PrP^C^ function(s) in neurons following the conversion of PrP^C^ into PrP^Sc^ [9–12].

From a physiological point of view, by acting as a signaling and/or a scaffolding molecule, PrP^C^ plays a central role in neuritogenesis able to promote the sprouting, outgrowth and maintenance of neurites [13,14]. PrP^C^ involvement in the very initial phase of neuritogenesis is supported by the observation that siRNA-mediated PrP^C^ silencing in 1C11 neuronal stem cells or PC12 cells (PrP^null^-cells) impairs neurite sprouting accompanying neuronal differentiation [15]. This PrP^C^ role relies on its capacity to control the signaling activity of plasma membrane β1 integrins, the downstream activity of RhoA-associated coiled-coil containing kinases (ROCK) and the dynamics of actin microfilaments [15]. In the absence of PrP^C^, overactivated ROCK reduces the turnover of actin fibers and exerts a dominant negative effect on the sprouting of neurites [15]. In differentiating and mature neurons, PrP^C^ influences neurite outgrowth and maintenance as well as synapse connectivity through its interaction with a set of diverse partners (N-CAM, STI-1, laminin γ-1, mGluR1-5, α7-nAChR) depending on the neuronal type [16–21] and the fine-tuning of Rho-GTPase and ROCK activities [14]. Besides, because neuritogenesis is intimately linked to the expression of neuronal functions, ROCK may hence take part to the onset, regulation and integration of neurotransmitter-associated functions. Whether PrP^Sc^-mediated corruption of the functional relationship between PrP^C^ and ROCK accounts for neuronal polarity alterations and abnormal neuronal functions, and contributes to TSEs progression, remains unknown.

To address these issues, we mainly exploit the properties of the 1C11 neuroectodermal cell line, which is endowed with the capacity to develop neurites and to acquire all functional properties of serotonergic neurons (1C11^5-HT^) within four days upon appropriate induction [22], and is chronically infected by mouse-adapted prions derived from scrapie (22L) or a human familial prion disease (Fukuoka-1, Fk) [23]. We previously reported that prion infection disturbs all neurotransmitter-associated functions in prion-infected 1C11^5-HT^ neuronal cells and triggers the production of neurotoxins, *i*.*e*. oxidative derivatives of serotonin [23]. In this study, we also take advantage of primary cultures of mature cerebellar granule neurons and of a cortico-striatal neuronal network reconstructed on microfluidic chips to assess the impact of prion infection on synapse connectivity and neurite integrity. We show here that prion infection impairs the development of neurites in the 1C11 neuronal stem cell line and triggers synapse disconnection and neurite degeneration in primary cultures of neurons. Defects in neuronal polarity originate from prion-induced overactivation of ROCK activity. Antagonizing ROCK activity rescues neurite sprouting of prion-infected 1C11 cells and protects prion-infected mature neurons from neurite degeneration. The beneficial effect afforded by ROCK inhibition on neuronal polarization is associated with restoration of neurotransmitter-associated functions and decrease of serotonin-derived neurotoxins levels.

Importantly, ROCK overactivation contributes to TSEs pathogenesis by stimulating the conversion of PrP^C^ into PrP^Sc^. We recently evidenced that PrP^Sc^ formation relates to a defect of cell surface TACE α-secretase neuroprotective activity towards PrP^C^ caused by the overactivation of the 3-phosphoinositide-dependent kinase 1 (PDK1) [24]. We demonstrate here that ROCK interact with and phosphorylate PDK1 leading to PDK1 overactivation in prion-infected cells. Inhibition of ROCK disrupts the ROCK-PDK1 complex, lowers PDK1 activity, rescues TACE activity at the plasma membrane and induces strong reduction of PrP^Sc^ level in prion-infected neuronal cells. Finally, inhibiting ROCK in mouse models of prion infection mitigates prion diseases.

## Results

### Prion infection of 1C11 neuronal stem cells impairs the onset of neurites through a ROCK-dependent mechanism

**Fig 1:**
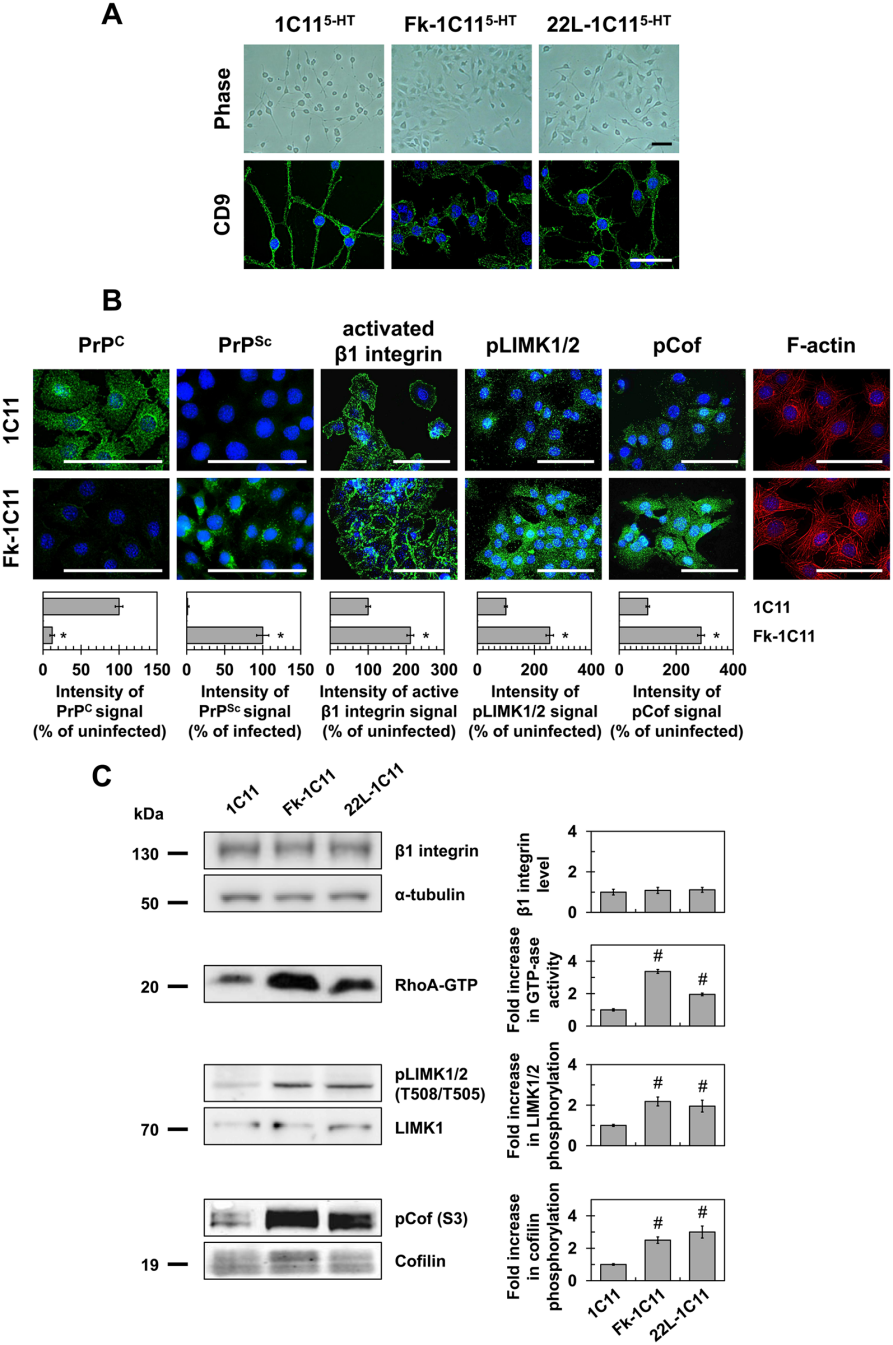
Prion infection of 1C11 neuronal stem cells alters neuritogenesis by overactivating the RhoA-ROCK-LIMK-cofilin pathway and modifying the actin network. (**A**) Phase pictures and cell contour staining with anti-tetraspanin CD9 antibody of Fk- or 22L-infected 1C11^5-HT^ cells as compared to uninfected neuronal 1C11^5-HT^ cells (day 4). Scale bars, 50 μm. (**B**) Immunofluorescent labeling of PrP^C^, PrP^Sc^ and activated β1 integrins at the surface of Fk-infected 1C11 cells as compared to their uninfected counterparts using SAF32, ICSM33 and 9EG7 antibodies, respectively. Immunofluorescent labeling of phospho-LIMK1/2 on Thr508/505 residues and phospho-cofilin on Ser3 and F-actin staining using TRITC-phalloidin within Fk-infected and uninfected 1C11 cells. Scale bars, 50 μm. (**C**) Western blots for total level of β1 integrins, phosphorylated LIMK1/2 and phosphorylated cofilin in 1C11 and Fk-1C11 cells. RhoA GTPase activity measured by pull-down assay in 1C11 and Fk-1C11 cells. Quantitative data are shown as the mean ± s.e.m. * *P* < 0.01 versus uninfected cells. # *P* < 0.05 versus uninfected cells.

When Fk- or 22L-infected 1C11 precursor cells were induced to differentiate towards the serotonergic program, less than 20% of Fk- and 22L-infected cells converted into neuronal-like cells with small, rounded cell bodies and bipolar extensions undistinguishable from uninfected 1C11^5-HT^ neuronal cells. The other 80% fraction did not adopt a neural-like morphology and/or presented spindle shaped cell bodies harboring wide and short extensions (Fig 1A).

These defects in neuronal polarization recall the impairment of neurite sprouting caused by PrP^C^ silencing in 1C11 progenitors and PC12 cells [15]. We previously showed that PrP^C^ depletion triggers the clustering and overactivation of β1 integrins, which in turn promote overactivation of the RhoA-ROCK-LIMK-cofilin pathway leading to alterations of F-actin architecture and subsequent gain of cell contractility [15]. In line with this, cell surface PrP^C^ immunolabeling under native conditions revealed that more than 90% of Fk-infected cells displayed very little fluorescence, while uninfected 1C11 cells stained brightly and uniformly (Fig 1B). This suggests a thorough conversion of cell surface PrP^C^ into PrP^Sc^ and *in fine* depletion of PrP^C^ molecules normally present at the plasma membrane of infected cells. PrP^Sc^ immunolabeling with PrP^Sc^ reacting ICSM33 antibody [25] under denatured conditions with guanidine thiocyanate indeed showed a high level of PrP^Sc^ in Fk-infected 1C11 cells (Fig 1B).

PrP^C^ conversion into PrP^Sc^ had no impact on total β1 integrin expression level as assessed by western blotting using the anti-CD29 antibody (Fig 1C). Nevertheless, cell surface immunofluorescence analyses using a β1 integrin antibody (9EG7) specifically targeting activated β1 integrins revealed that the pool of activated integrins was ~2-fold increased in Fk-infected cells as compared to uninfected 1C11 cells (Fig 1B). In addition, active β1 integrins were evenly distributed and displayed a dot-like staining at the cell periphery of uninfected 1C11 cells, while they clustered and formed elongated patches in Fk-infected 1C11 cells (Fig 1B).

As compared to uninfected cells, we measured increases by 2- to 3-fold in RhoA GTPase activity (Fig 1C), the phosphorylation level of LIMK1/2 on Thr505 and Thr508 (Fig 1B and 1C), and the phosphorylation level of cofilin on Ser3 (Fig 1B and 1C) in Fk- and 22L-infected 1C11 cells, indicating that prion infection reduces cofilin-associated severing activity towards F-Actin [26–29]. Accordingly, fibrillar actin (F-actin) microfilaments were large and disorganized in the cytoplasm of Fk-infected 1C11 cells, while F-actin distributed as thin, parallel stress fibers underneath the cell plasma membrane of uninfected 1C11 precursor cells (Fig 1B).

These overall data show that prion infection triggers overactivation of the RhoA-ROCK-LIMK-cofilin signaling pathway, which alters the architecture and turnover of F-actin microfilaments, thereby contributing to neuritogenesis impairment.

### ROCK inhibition in prion-infected 1C11 cells restores neuritogenesis and rescues overall neurotransmitter-associated functions

**Fig 2:**
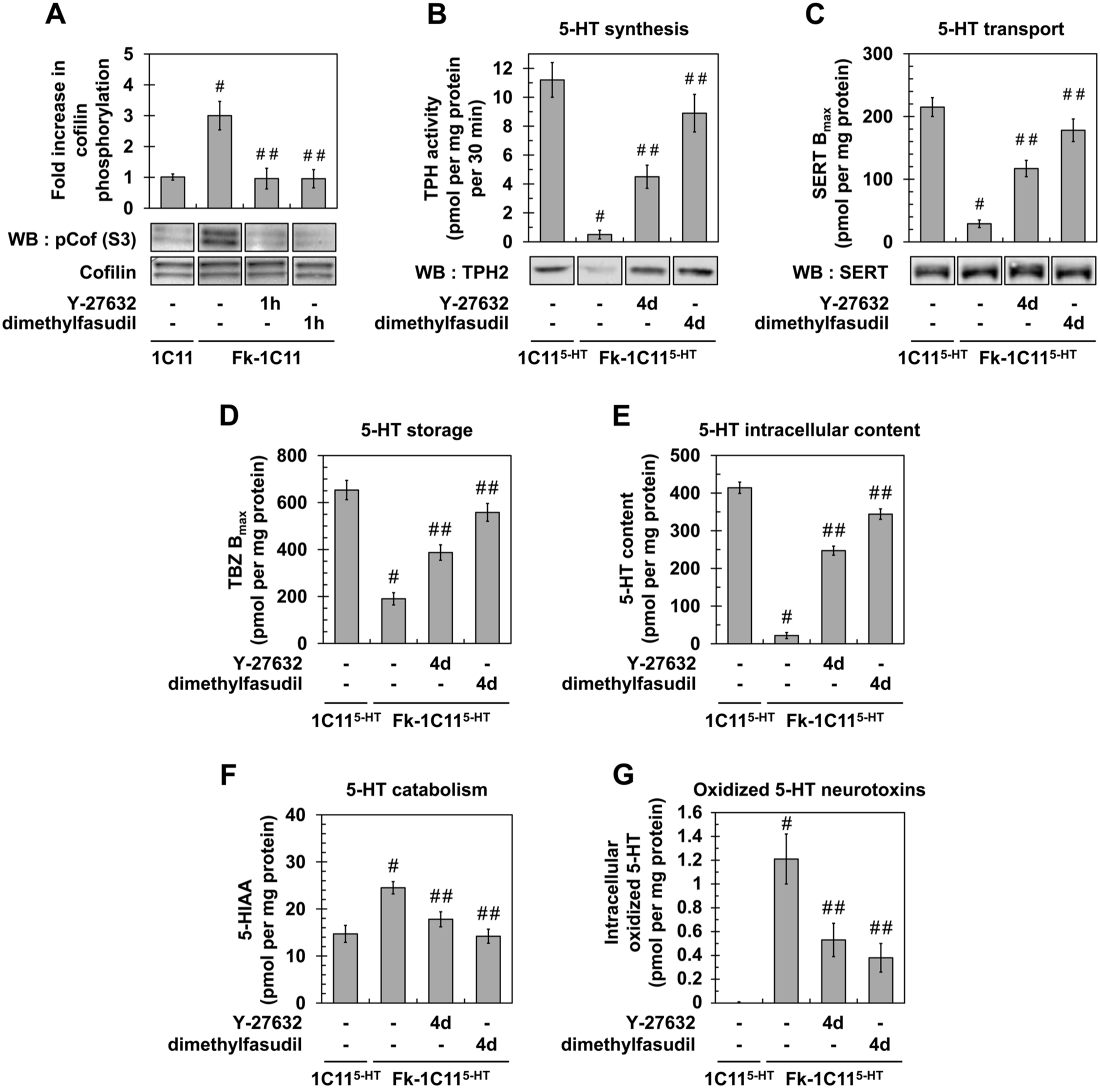
Inhibition of ROCK restores neurotransmitter-associated functions in prion-infected 1C11^5-HT^ neuronal cells. (**A**) Western blot and histogram quantifications for phosphorylated cofilin on Ser3 in Fk-infected 1C11 cells treated or not with dimethylfasudil (2 μM) or Y-27632 (100 μM) for 1h versus uninfected 1C11 cells. (**B**) 5-HT synthesis (TPH activity) and TPH immunoblotting, (**C**) transport by SERT (paroxetine binding) and SERT immunoblotting, (**D**) storage (VMAT-2 tetrabenazine binding), (**E**) intracellular content, and (**F**) catabolism (5-HIAA concentration) in uninfected 1C11^5-HT^ cells and Fk-infected 1C11^5-HT^ cells differentiated for 4 days along the serotonergic pathway in the absence or presence of Y-27632 (100 μM) or dimethylfasudil (2 μM). (**G**) Concentration of 5-HT-derived oxidized species in cell lysates of 1C11^5-HT^ cells and Fk-1C11^5-HT^ differentiated in the absence or presence of Y-27632 or dimethylfasudil. n = 6 for each condition. Values are the mean ± s.e.m. # *P* < 0.05 versus uninfected cells. ## *P* < 0.05 versus infected cells.

We next wondered whether inhibiting ROCK activity would counteract prion-induced neuritogenesis defects. When treated with two distinct ROCK inhibitors, namely Y-27632 (100 μM) or dimethylfasudil (2 μM) (for review see [30–32] and references therein), ~90% of Fk- or 22L-infected 1C11 cells induced to differentiate along the serotonergic pathway developed neurites. At the end of the program (day 4), prion-infected 1C11 cells differentiated in the presence of ROCK inhibitors (referred to as Rocki-infected 1C11^5-HT^ cells) exhibited neurites that were thin, bipolar and extended from the ovoid cell body as for uninfected 1C11^5-HT^ neuronal cells (S1 Fig and S1 Table). The mean neurite length of Rocki-infected 1C11^5-HT^ cells was comparable to that of uninfected 1C11^5-HT^ cells differentiated in the presence of ROCK inhibitors (S1 Table). Rescued neuritogenesis by Fk-infected 1C11 precursor cells upon ROCK inhibition was associated with decrease to basal level of phospho-cofilin level on Ser 3 (Fig 2A), which restores cofilin severing activity towards F-actin and renders Fk-1C11 precursor cells competent to sprout neurites when induced towards the neuronal program [15].

Beyond the rescue of neuronal polarity, inhibition of ROCK restores the overall neuronal functions, *i*.*e*. synthesis, storage, degradation and transport of 5-HT, in Rocki-infected 1C11^5-HT^ cells. While the activity of tryptophan hydroxylase (TPH), the 5-HT synthesizing enzyme, in Fk-1C11^5-HT^ cells represented 5% of that measured with uninfected 1C11^5-HT^ cells, TPH activity increased by 8- to 18-fold in Rocki-Fk-1C11^5-HT^ cells treated with Y-27632 or dimethylfasudil, respectively (Fig 2B). Corroborating TPH activity, western blot analysis revealed that the TPH2 enzyme was weakly present in Fk-1C11^5HT^ cells compared to uninfected 1C11^5-HT^ cells, while TPH2 expression was recovered in Rocki-Fk-1C11^5-HT^ cells (Fig 2B). This indicates that PrP^Sc^-induced ROCK overactivation impairs TPH protein synthesis.

Using the paroxetine Serotonin Re-uptake Inhibitor (SRI) antidepressant to count functional Serotonin Transporter (SERT) molecules [33], we showed that the number of functional SERT proteins was 5- (Y-27632) to 7- (dimethylfasudil) fold enhanced in Rocki-Fk-1C11^5-HT^ cells as compared to Fk-1C11^5-HT^ cells (where functional SERT level was 10% of that measured with uninfected 1C11^5-HT^ cells) (Fig 2C), indicating that inhibition of ROCK rescues SERT functionality. By contrast with TPH2 enzyme, no significant variation in SERT protein level could be evidenced between Fk-1C11^5-HT^, Rocki-Fk-1C11^5-HT^ and uninfected 1C11^5-HT^ cells (Fig 2C). This indicates that the impact of prion infection on SERT activity does not relate to modulation of SERT synthesis.

Using [^3^H]-tetrabenazine that selectively binds to the Vesicular Monoamine Transporter (VMAT), we monitored that antagonizing ROCK activity restored 5-HT storage in Rocki-Fk-1C11^5-HT^ cells with a tetrabenazine binding value that was 2- (Y-27632) to 2.5- (dimethylfasudil) fold increased as compared to Fk-infected 1C11^5-HT^ cells (30% of that measured with uninfected 1C11^5-HT^ cells) (Fig 2D). Restoration of normal serotonergic neuronal functions upon ROCK inhibition was further supported by a 10- (Y-27632) to 15- (dimethylfasudil) fold increase of 5-HT content (Fig 2E), decreased levels (30 to 40%) of the 5-hydroxyindolacetic acid (5-HIAA) degradative product of 5-HT (Fig 2F), and deep reduction (50 to 70%) in the intracellular content of serotonin-derived oxidized neurotoxins (Fig 2G).

Altogether, these data demonstrate that inhibition of ROCK activity in prion-infected 1C11 cells rescues neuritogenesis and the overall serotonergic-associated functions.

### Prion infection of primary neurons triggers synapse disconnection and neurite degeneration through a ROCK-dependent mechanism

To extend our study to mature neurons, we combined the use of primary cultures of mouse cerebellar granule neurons (CGNs) and of a reconstructed cortico-striatal neuronal network grown on microfluidic chips [34–36]. CGNs are helpful to probe the effect of prion infection on the axon and dendrites [37,38]. The reconstructed cortico-striatal network on microfluidic device offers the possibility to infect cortical neurons in the somato-dendritic side and to monitor the impact of prion infection at distance on synapse connectivity between cortical and striatal neurons.

**Fig 3:**
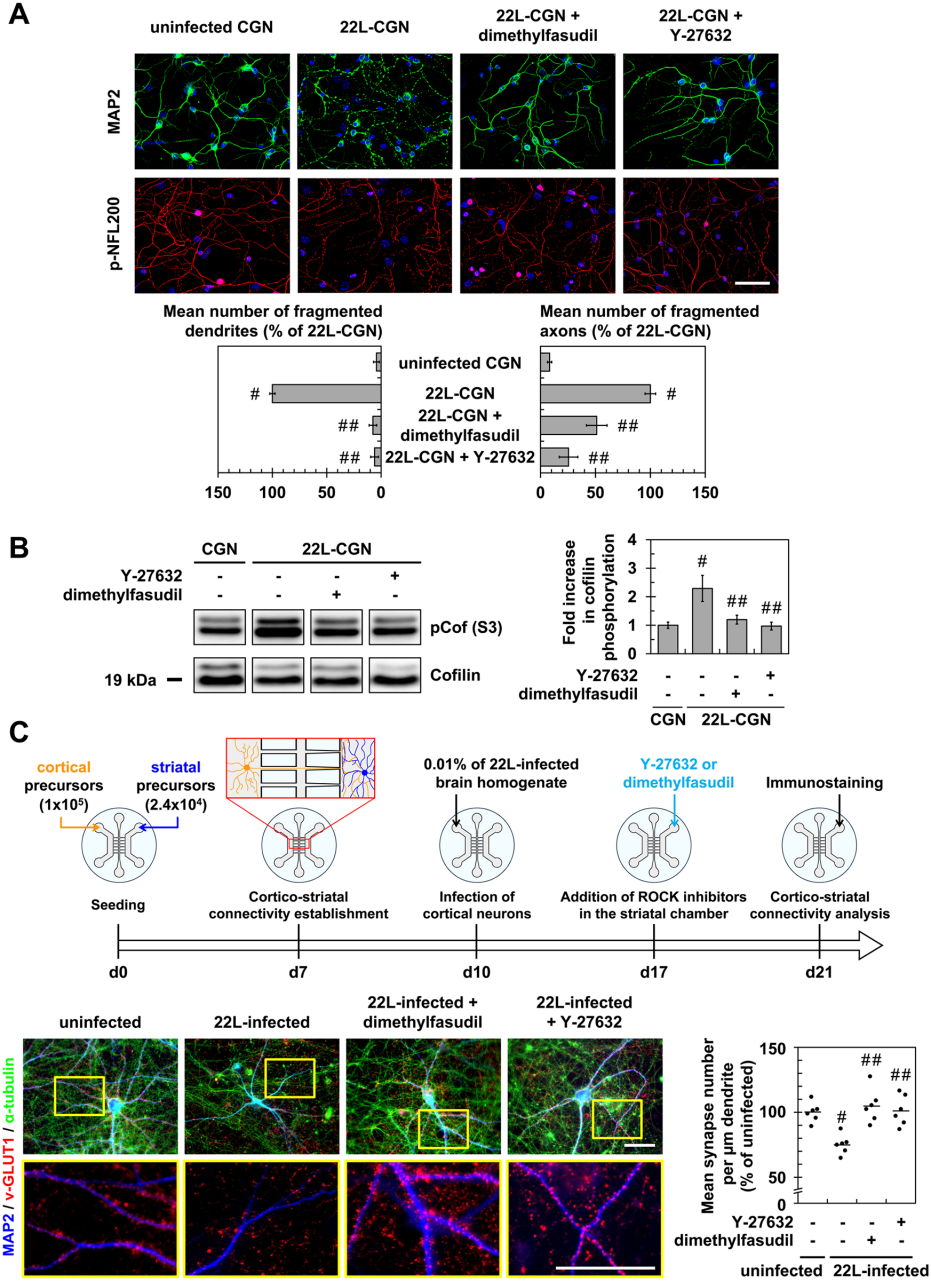
Inhibition of ROCK protects primary neuronal cultures from prion-induced synapse disconnection and neurite degradation. (**A**) Immunofluorescent labeling of dendrites (anti-MAP2 staining, green) and axons (phospho-NFL200 staining, red) of cerebellar granule neurons (CGNs) infected with 22L prions for 7 days and then treated 4 days with Y-27623 (100 μM) or dimethylfasudil (2 μM) as compared to uninfected CGNs. Scale bars, 50 μm. Quantification histograms of neurons with fragmented dendrites or axons. (**B**) Western blot and histogram quantifications for phosphorylated cofilin on Ser3 in 22L-infected CGNs treated or not with dimethylfasudil or Y-27632 versus uninfected CGNs. (**C**) Schematic of the experimental procedure with a reconstructed cortico-striatal neuronal network on microfluidic devices and immunofluorescent labelings. Cortical neurons of a 10 days-*in-vitro* (DIV)-aged cortico-striatal network were infected with 22L prions for 7 days. Striatal neurons were then treated with Y-27632 (20 μM) or dimethylfasudil (2 μM) for 4 days. Dendrites (anti-MAP2 staining, blue) of striatal neurons and synapses (anti-v-GLUT1 staining, red) and axons (anti-α-tubulin, green) of cortical neurons were labeled with specific antibodies. Close proximity of blue (striatal dendrites) and red (v-GLUT1 cortical presynaptic terminal) signals indicates cortico-striatal connectivity. Scale bars, 50 μm. Each point corresponds to the mean of number synapses per μm dendrite determined from the analysis of ten pictures. n = 6 for each condition. For each group, individual mean values and mean (line) are shown. # *P* < 0.05 versus uninfected cells. ## *P* < 0.05 versus non treated infected cells.

We showed that infection of CGNs with 22L strain triggered neuronal dysfunction as inferred by the fragmentation of axon and dendrites by 11 days post-infection (dpi), using SMI31 and MAP2 as markers of the axon and dendrites, respectively (Fig 3A). Western blot analyses revealed a 2-fold increase in cofilin phosphorylation level in 22L-infected CGNs *vs*. uninfected CGNs (Fig 3B), indicative of an overactivation of ROCK in infected CGNs. Treatment of 22L-infected CGNs with the ROCK inhibitors Y-27632 (100 μM) or dimethylfasudil (2 μM) at 7 dpi for 4 days exerted a protective effect towards PrP^Sc^-induced neurite degeneration, since ~70 to 90% of treated infected neurons displayed unfragmented dendrites and axon (Fig 3A). Such protective effect of ROCK inhibitors on axon and dendrites of 22L-infected CGNs relates to rescued cofilin activity as shown by cofilin phosphorylation level that returned to basal level upon cell treatment with either Y-27632 or dimethylfasudil (Fig 3B).

With reconstructed cortico-striatal networks, we observed that challenging the cortical compartment with 22L strain promoted disconnection and retraction of synapses between cortical and striatal neurons by 11 dpi, as inferred by pre-synaptic cortical v-GLUT1 immunostaining no longer colocalized with post-synaptic striatal dendrite MAP2 staining (Fig 3C). ROCK inhibition with Y-27632 (20 μM) or dimethylfasudil (2 μM) applied in the striatal chamber by 7 dpi for 4 days attenuated PrP^Sc^-induced neuronal disconnection and synapse retraction by ~100% (Fig 3C).

As a whole, our data indicate that prion-induced ROCK activation in mature neurons disrupts neuronal polarity and connectivity. Inhibition of ROCK activity protects neurons from prion-induced synapse disconnection and dendrite/axon degeneration.

### ROCK inhibition promotes strong reduction of PrP^Sc^ level by rescuing TACE α-secretase-mediated PrP^C^ α-cleavage in a PDK1-dependent manner

**Fig 4:**
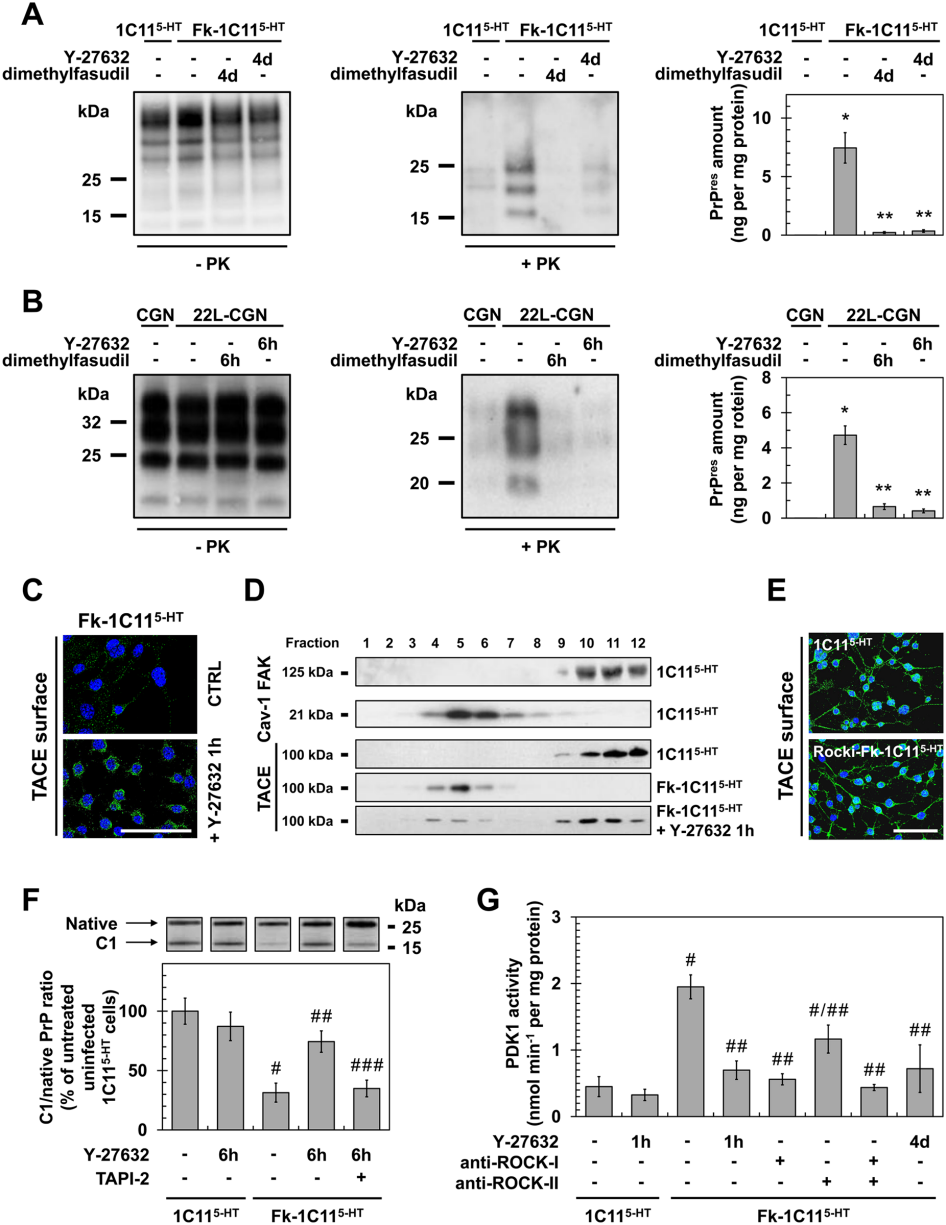
ROCK inhibition lowers PrP^Sc^ level by rescuing TACE α-secretase neuroprotective activity towards PrP^C^ in a PDK1-dependent manner. (**A, B**) Amount of PrP^Sc^ in Rocki-Fk-1C11^5-HT^ cells differentiated into serotonergic neuronal cells for 4 days (4d) in the presence of Y-27632 (100 μM) or 22L-infected CGNs exposed for 6 h to Y-27632 (100 μM), as shown by western blotting (left) and quantified by ELISA (right). n = 10 for each condition. (**C**) Immunofluorescent labeling of TACE at the surface of Fk-infected 1C11^5-HT^ cells treated or not with the ROCK inhibitor (Y-27632, 100 μM) for 1 h. Scale bar, 50 μm. (**D**) Immunoblot analysis of sucrose gradient fractions of membranes of Fk-infected 1C11^5-HT^ cells treated or not with Y-27632 (100 μM, 1h) versus uninfected 1C11^5-HT^ to assess TACE displacement from the plasma membrane (FAK-enriched fractions) to caveolin-1-enriched vesicles. FAK = Focal Adhesion Kinase. Cav-1 = caveolin-1. (**E**) Immunofluorescent labeling of TACE at the surface of Rocki-Fk-1C11^5-HT^ differentiated for 4 days in the presence of Y-27632 (100 μM) versus uninfected 1C11^5-HT^ cells. Scale bar, 50 μm. (**F**) Western blot analysis (top) of the C1 fragment of PrP (C1) and full-length PrP (native) in Fk-infected 1C11^5-HT^ cells treated or not with Y-27632 (100 μM) or a combination of Y-27632 (100 μM) and TAPI-2 (100 μM) for 6 h versus uninfected cells and the ratio (bottom) of C1/native full-length PrP. n = 5 for each condition. (**G**) PDK1 activity in Fk-infected 1C11^5-HT^ cells treated or not with Y-27632 (100 μM, 1 h), in Fk-infected 1C11^5-HT^ bombarded with tungsten microprojectiles coated with ROCK-I or ROCK-II antibodies or both, or in Rocki-Fk-1C11^5-HT^ cells differentiated for 4 days in the presence of Y-27632 (100 μM) versus uninfected 1C11^5-HT^ cells. n = 5 for each condition. Values are the mean ± s.e.m. for all experiments. * *P* <0.01 versus uninfected cells. ** *P* <0.01 versus non-treated infected cells. # *P* <0.05 versus uninfected cells. ## *P* <0.05 versus non treated infected cells. ### *P* <0.05 versus infected cells treated with Y-27632.

We next investigated whether PrP^Sc^ accumulation in prion-infected cells would depend on ROCK overactivity. Inhibition of ROCK with dimethylfasudil (2 μM) or Y-27632 (100 μM) decreased the amount of proteinase K-resistant PrP^Sc^ (PrP^res^) by 80 to 90% in either Rocki-Fk-1C11^5-HT^ cells (Fig 4A) or primary cultures of 22L-infected CGNs (Fig 4B). These observations introduce ROCK overactivation as a novel pathogenic event contributing to the conversion of PrP^C^ into PrP^Sc^.

We recently reported that accumulation of PrP^Sc^ in prion diseases relates to internalization of TACE α-secretase in caveolin-1 (Cav-1)-enriched microvesicles caused by PDK1 overactivation, which cancels PrP^C^ neuroprotective α-cleavage by TACE [24]. The question was thus to assess whether the reduction of PrP^Sc^ level measured upon ROCK inhibition would depend on a control of the PDK1-TACE module by ROCK. Inhibition of ROCK with Y-27632 (100 μM, 1h) promoted translocation of TACE back to the cell surface of Fk-1C11^5-HT^ cells (Fig 4C) and 22L-infected CGNs (S2A Fig). Detergent-free sucrose gradient membrane fractionation of cell extracts followed by western blotting further revealed that TACE no longer co-distributed with Cav-1 in Fk-1C11^5-HT^ cells exposed to Y-27632, but was found together with the focal adhesion kinase (FAK) at the plasma membrane of infected 1C11^5-HT^ cells as observed with uninfected 1C11^5-HT^ cells (Fig 4D). With Rocki-Fk-1C11^5-HT^ cells differentiated for 4 days in the presence of Y-27632, TACE was at the plasma membrane of both the cell body and neurites with a distribution pattern highly comparable to that of uninfected 1C11^5-HT^ cells (Fig 4E).

Redirection of TACE to the cell surface of infected cells upon ROCK inhibition rescued TACE-mediated α-cleavage of PrP between residues 111/112 and generated an N-terminal truncated fragment (that is, membrane C1) (Figs 4F and S2B) that does not convert into PrP^Sc^ [39]. In Fk-1C11^5-HT^ cells and 22L-CGNs, the ratio between PrP C1 fragment and full length PrP (native) was reduced by approximately 75% as compared to that of uninfected cells (Figs 4F and S2B), thus accounting for conversion of PrP^C^ into PrP^Sc^. In infected cells treated with Y-27632 (100 μM) for 6 h the C1/native ratio was ~2- to 3-fold increased compared to untreated infected cells (Figs 4F and S2B), indicative of restored PrP^C^ α-cleavage. Such an increase in C1/native ratio in the presence of the ROCK inhibitor depends on TACE activity, since inhibition of TACE with TAPI-2 (100 μM, 1h) counteracted the effects of Y-27632 on PrP neuroprotective cleavage (Figs 4F and S2B).

Finally, while PDK1 activity was 2- to 3-fold increased in Fk-infected 1C11^5-HT^ cells (Fig 4G) and 22L-infected CGNs (S2C Fig), ROCK inhibition in Fk-1C11^5-HT^ cells or 22L-CGNs with Y-27632 (100μM, 1h) reduced PDK1 activity by ~50 to 70% to basal level measured with uninfected cells (Figs 4G and S2C). Of note, bombardment of Fk-infected 1C11^5-HT^ cells with tungsten microprojectiles coated with ROCK-I antibodies [24,40] restored basal PDK1 activity, while tungsten microprojectiles coated with ROCK-II antibodies triggered less reduction of PDK1 activity (~45%) (Fig 4G), indicating that ROCK-I overactivity within an infectious context mainly contributes to the overactivation of PDK1. The rise in PDK1 activity was also counteracted in Rocki-Fk-1C11^5-HT^ cells differentiated for 4 days in the presence of Y-27632 (Fig 4G), further demonstrating that the control of PDK1 activity by ROCK-I occurs in polarized cells as well as in non-polarized Fk-1C11^5-HT^ cells.

These overall data firmly establish that ROCK act as upstream positive regulators of PDK1 activity and contribute to prion-induced neurodegeneration by favoring the production of PrP^Sc^.

### ROCK-I interacts with PDK1 and promotes PDK1 phosphorylation

**Fig 5:**
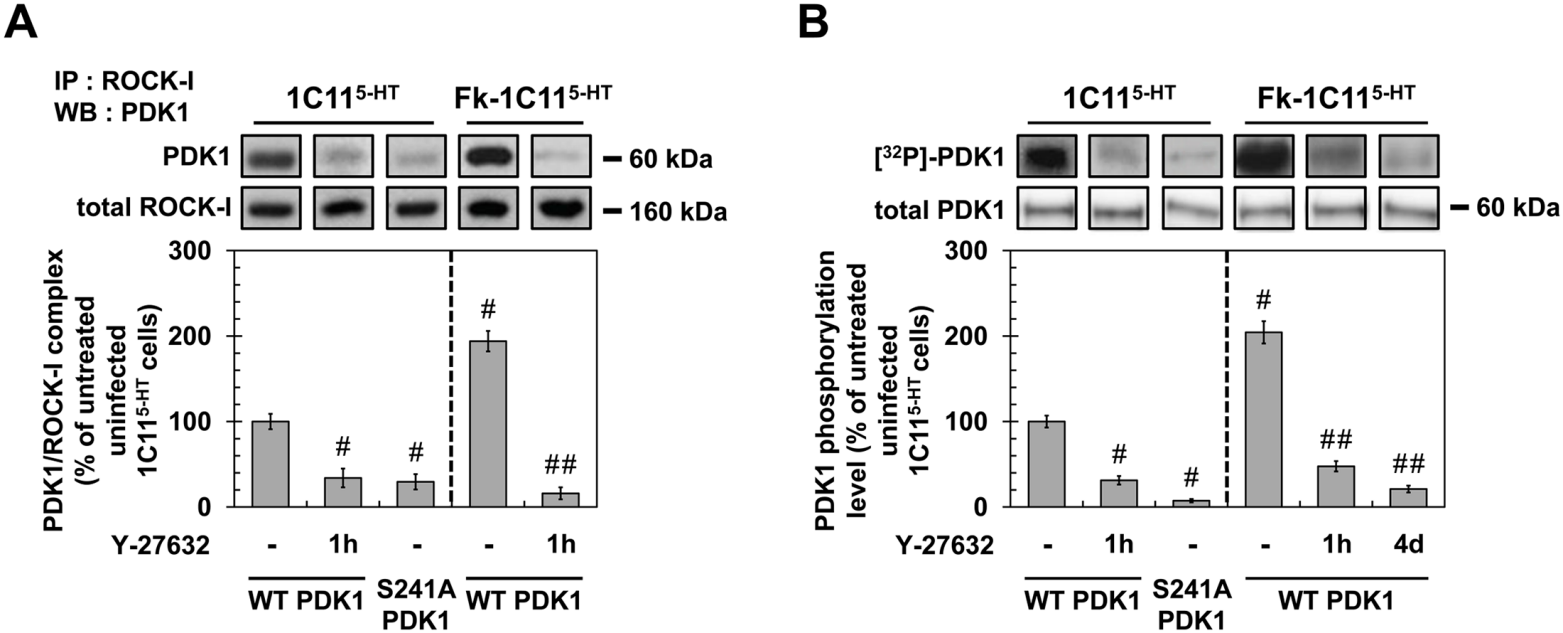
ROCK-I interacts with PDK1 and phosphorylates PDK1. (**A**) Immunoprecipitation of ROCK-I and immunoblotting of PDK1 in uninfected 1C11^5-HT^ or Fk-1C11^5-HT^ treated or not with Y-27632 (100 μM, 1h) as well as in 1C11^5-HT^ transfected with S241A PDK1 mutant. (**B**) Cell ^32^P metabolic labeling followed by PDK1 immunoprecipitation and western blotting for PDK1 phosphorylation level in uninfected 1C11^5-HT^ or Fk-1C11^5-HT^ treated or not with Y-27632 (100 μM, 1h) as well as in 1C11^5-HT^ transfected with S241A PDK1 mutant. Values are the mean ± s.e.m. # *P* < 0.05 versus non treated uninfected cells. ## *P* < 0.05 versus non treated infected cells.

The acute regulation of PDK1 activity relies on a complex interplay between PDK1 phosphorylation, subcellular localization, binding of regulators and conformational changes (for review see [41] and references therein). To investigate how ROCK-I regulates PDK1 activity, we designed a first set of experiments centered on the interaction between ROCK-I and PDK1. In uninfected 1C11^5-HT^ cells, ROCK-I immunoprecipitation followed by PDK1 western blotting revealed that ROCK-I interacts with PDK1 (Fig 5A). In Fk-infected 1C11^5-HT^ cells, the fraction of PDK1 interacting with ROCK-I was ~2-fold increased compared to uninfected cells (Fig 5A). Of note, the level of ROCK-I did not vary between uninfected 1C11^5-HT^ and Fk-1C11^5-HT^ cells (Fig 5A). The rise in ROCK activity induced by PrP^Sc^ thus increases the number of PDK1 molecules recruited by ROCK-I. Exposure of uninfected 1C11^5-HT^ or Fk-1C11^5-HT^ cells to Y-27632 (100 μM) for 1h dissociated the ROCK-I / PDK1 complex by ~70% and ~90%, respectively (Fig 5A), further indicating that the ROCK-I-kinase activity is necessary for its association with PDK1.

To next probe whether ROCK-I phosphorylates PDK1, cells treated or not with Y-27632 (100 μM, 1h) were metabolically labeled for 60 min with [^32^P]-orthophosphate (8.81 μCi.mL^−1^) and [^32^P]-labeled PDK1 level was quantified after PDK1 immunoprecipitation and western blotting. In uninfected 1C11^5-HT^ cells, PDK1 phosphorylation level was reduced by ~70% in the presence of Y-27632 (Fig 5B), indicating that ROCK-I does phosphorylate PDK1 under physiological conditions. From a mechanistic point of view, autophosphorylation of PDK1 Ser241 is critical but not sufficient to induce PDK1 full activity [42]. Depending on the cell type and signaling pathways converging on PDK1, additional phosphorylations on Ser, Thr and Tyr residues located in the kinase domain, the pleckstrin-homology (PH) domain and in the linker between the kinase and PH domains have already been shown to enhance PDK1 activity in a more or less cooperative manner [41,43]. Here, we provide evidence that PDK1 autophosphorylation at Ser241 precedes PDK1 phosphorylation by ROCK-I on yet-to-be identified residues. Indeed, site-directed mutagenesis of PDK1 Ser241 into Ala to mimic a constitutive dephosphorylation (S241A) state decreased by ~90% the incorporation of ^32^P on the S241A PDK1 mutant when transfected into 1C11^5-HT^ cells (Fig 5B). Besides, immunoprecipitation experiments showed that ROCK-I did not complex with the S241A PDK1 mutant (Fig 5A). These data establish that autophosphorylation of PDK1 Ser241 is essential for PDK1 interaction with ROCK and further phosphorylation by ROCK.

We next examined the phosphorylation status of PDK1 within an infectious context. The level of phosphorylated PDK1 was 2-fold higher in Fk-infected 1C11^5-HT^ cells than in uninfected cells (Fig 5B). Such increase in PDK1 phosphorylation level was cancelled upon treatment of Fk-1C11^5-HT^ cells with Y-27632 (100 μM) for 1h or in Rocki-Fk-1C11^5-HT^ cells left to differentiate for 4 days in the presence of Y-27632 (Fig 5B), indicating that prion-induced ROCK overactivity enhances PDK1 phosphorylation level and that ROCK-induced phosphorylation of PDK1 is independent on the acquisition of neuronal polarity.

As a whole, our data reveal that ROCK-I constitutively complexes with and phosphorylates PDK1 in uninfected cells. Autophosphorylation of PDK1 at Ser241 is a prerequisite for PDK1 interaction with ROCK-I and further phosphorylation by ROCK-I. In prion-infected cells, ROCK overactivation augments by 2-fold the pool of PDK1 molecules interacting with and phosphorylated by ROCK-I, which thus enhances PDK1 activity.

### Antagonizing ROCK activity improves disease in mouse models of prion infection

**Fig 6:**
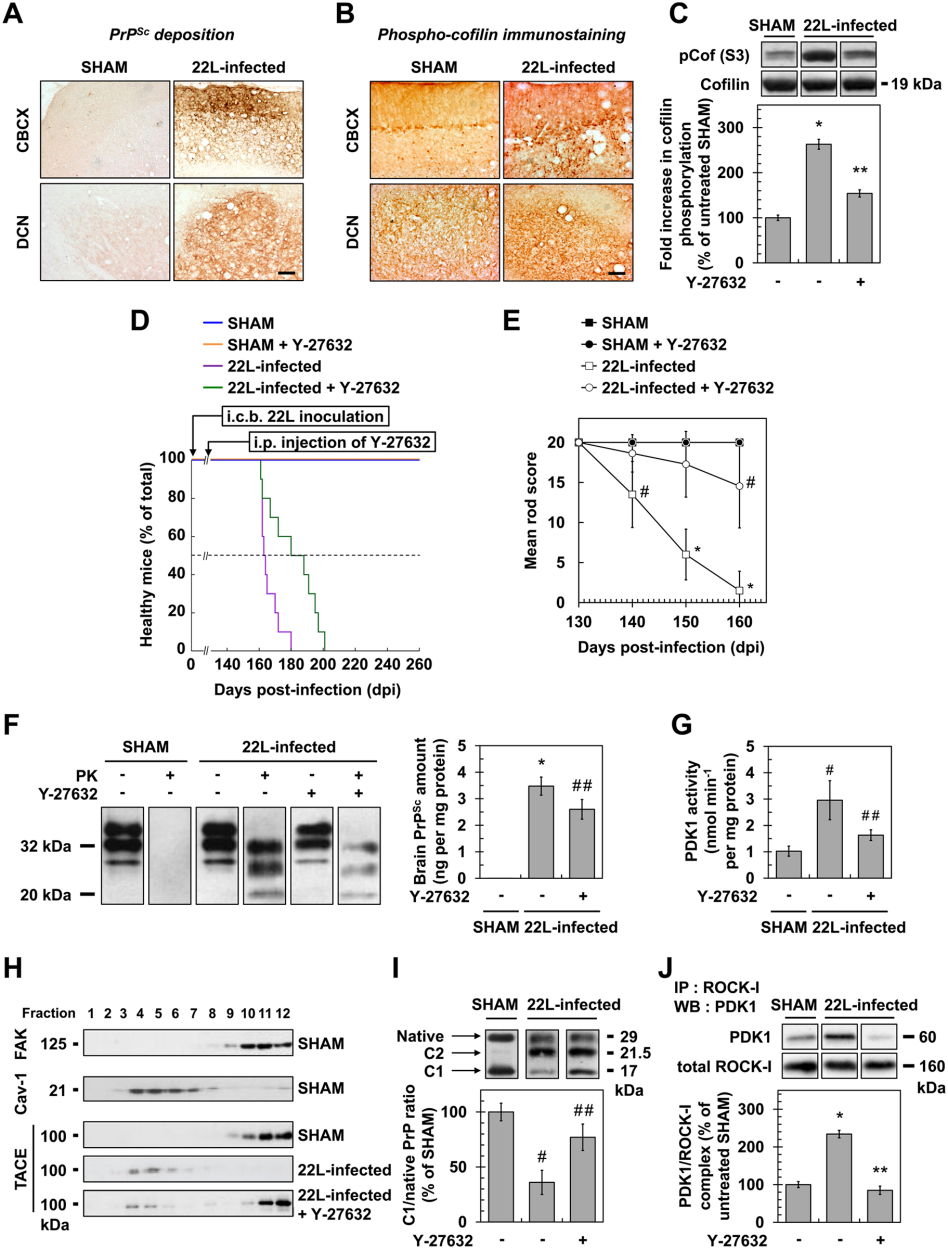
ROCK inhibition with Y-27632 attenuates prion disease in mice. (**A**) Cerebellar immunoperoxidase staining to visualize PrP^Sc^ deposition in the cerebellar cortex (CBCX) and deep cerebellar nuclei (DCN) of 22L-infected mice, Scale bars, 100 μm. (**B**) Immunoperoxidase staining to visualize phosphorylated cofilin on Ser3 in CBCX and DCN of mock-inoculated (SHAM) and 22L-infected mice. Scale bars, 100 μm. (**C**) Western blot and histogram quantifications for phosphorylated cofilin on Ser3 in 22L-infected mice infused or not with Y-27632 versus SHAM mice. n = 4 in triplicate. (**D**) Survival curves of SHAM and 22L-inoculated mice via the intracerebellar route (i.c.b.) infused or not with the ROCK inhibitor Y-27632 by intraperitoneal injection (i.p.) starting at 130 days after infection (5 mg per kg body weight per day; 0.25 μl h^-1^). n = 10 mice per group. (**E**) Static rod test between 130 and 160 days after infection in 22L-infected mice treated with Y-27632. n = 10 mice per group. (**F**) Left, Western-blot for proteinase K-resistant PrP^Sc^ in brain extracts from SHAM and 22L-infected mice infused or not with Y-27632. Right, post-mortem quantification of proteinase K-resistant PrP^Sc^ in brains of 22L-infected mice treated or not with Y-27632. n = 10 for each condition. (**G**) PDK1 activity in cerebellar extracts of 22L-infected mice treated or not with Y-27632 versus SHAM mice. n = 9 for each condition. (**H**) Immunoblot analysis of sucrose gradient fractions of cerebellar extracts of SHAM mice and 22L-infected mice infused or not with Y-27632 to assess TACE displacement from the plasma membrane (FAK-enriched fractions) to caveolin-1-enriched vesicles *in vivo*. (**I**) Western blot analysis (top) of the C1 fragment of PrP (C1) and full-length PrP (native) in cerebellar extracts from SHAM and 22L-infected mice infused or not with Y-27632 and the ratio (bottom) of C1/native full-length PrP. Note that mouse infection with 22L strain is associated with decreased PrP α-cleavage at the expense of PrP β-cleavage that generates C2 fragment [68, 69]. n = 5 for each condition. (**J**) ROCK immunoprecipitation followed by PDK1 western blotting in cerebellar extracts of 22L-infected mice treated or not with Y-27632 versus SHAM mice. n = 9 for each condition. Values are the mean ± s.e.m. * *P* < 0.01 versus SHAM mice. ** *P* < 0.01 versus 22L-infected mice. # *P* < 0.05 versus SHAM mice ## *P* < 0.05 versus 22L-infected mice.

In adult C57BL/6J mice inoculated with the mouse-adapted scrapie strain 22L via the intracerebellar route [44] and sacrificed at 130 days post infection (dpi) just before the symptomatic phase, a marked increase in phosphorylated cofilin immunostaining that matched with PrP^Sc^ deposition was observed in the brain of prion-infected mice in both the cerebellar cortex (CBCX) and deep cerebellar nuclei (DCN) compared to uninfected animals (SHAM) (Fig 6A and 6B). Western-blot analyses of cerebellum extracts indicated a ~2 to 3-fold increased level of phosphorylated cofilin in 22L-infected *vs*. SHAM mice (Fig 6C).

Because increased cofilin phosphorylation in the brain of prion-infected mice argues for ROCK overactivity *in vivo*, we next wondered whether inhibition of ROCK would attenuate prion disease. Y-27632, known to easily cross the Blood Brain Barrier [30], was first chronically injected intraperitoneally (i.p.) in adult C57BL/6J mice starting 130 days after infection and before the onset of clinical signs (140 days). We showed that Y-27632 treatment delayed mortality in 22L-infected mice as compared to untreated mice (181.4 +/- 4.7 days versus 166.0 +/- 1.8 days, n = 10, *P* < 0.0001, Fig 6D) with no overt sign of Y-27632 toxicity. Reduction of phosphorylated cofilin level in post-mortem cerebellum extracts from 22L-infected mice upon Y-27632 treatment indicated ROCK inhibition in brain (Fig 6C). Y-27632 infusion also decreased prion infection-induced impairments in motor function (Fig 6E). While in 22L-infected mice the mean static rod score dropped to 10 by 145 days, in Y-27632-treated infected mice, the mean static rod score never dropped below 10. In SHAM mice, motor coordination was non-sensitive to Y-27632 treatment.

We further showed that the beneficial effect of ROCK inhibition against prion disease correlated with decreased levels of PrP^Sc^. Post-mortem quantifications of proteinase K-resistant PrP revealed a ~30% reduction in PrP^res^ level in the brains of 22L-infected mice infused with Y-27632 compared to untreated infected animals (Fig 6F), indicative of PDK1 down-regulation (Fig 6G), TACE relocation to the plasma membrane (Fig 6H) and rescue of PrP^C^ α-cleavage by TACE upon ROCK inhibition *in vivo* (Fig 6I). Accordingly, while PDK1 activity was ~3-fold enhanced in the brain of 22L-infected mice compared to SHAM mice, PDK1 activity decreased by ~50% in prion-infected mice treated with Y-27632 *vs*. untreated infected mice (Fig 6G). As for prion-infected 1C11^5-HT^ cells and CGNs, reduction of PDK1 activity in the brain of 22L-infected mice infused with Y-27632 originated from disruption of the ROCK-PDK1 complex (Fig 6J).

Finally, C57Bl/6J mice inoculated with 22L prions were also intraperitonealy injected with dimethylfasudil following the same procedure as for Y-27632. ROCK inhibition with dimethylfasudil prolonged the survival time of infected mice compared to untreated mice (174.8 +/- 4.3 days versus 166.0 +/- 1.8 days, n = 10, *P* < 0.0001, S3A Fig), reduced phospho-cofilin level in the cerebellum (S3B Fig), counteracted prion-induced motor deficits (S3C Fig), decreased brain PrP^Sc^ level by 30% (S3D Fig), as a consequence of dissociation of the ROCK/PDK1 complex (S3E Fig) and reduction of PDK1 activity (S3F Fig).

Altogether, these data demonstrate that targeting ROCK activity mitigates prion diseases.

## Discussion

**Fig 7:**
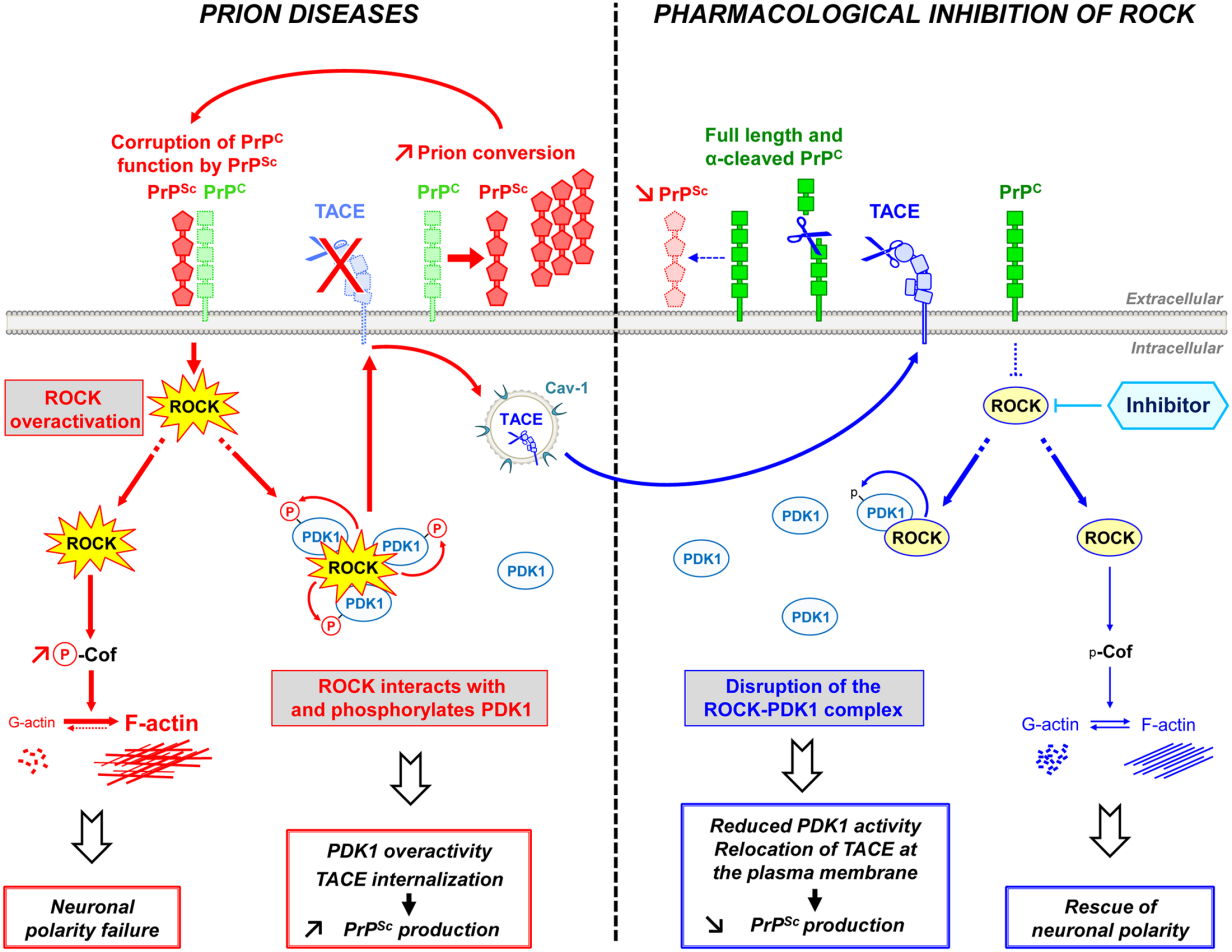
Schematic representation of ROCK dysregulation in prion-infected cells and incidence of ROCK overactivity on neuritogenesis and PrP^Sc^ production. Prion infection causes the overstimulation of ROCK. Overactivated ROCK alters neuronal polarity by disrupting the dynamics of F-actin cytoskeleton through the LIM kinases/cofilin signaling pathway. Overactivated ROCK also amplifies PrP^Sc^ production by acting on the PDK1/TACE signaling module. Prion infection increases the pool of PDK1 molecules interacting with ROCK and phosphorylated by ROCK, at the root of increased PDK1 activity. ROCK-induced PDK1 overactivation promotes the internalization of TACE α-secretase in caveolin-1-enriched vesicles, which cancels TACE neuroprotective α-cleavage of PrP^C^ at the plasma membrane of prion-infected cells. The accumulation of PrP^Sc^ fuels the activation of ROCK, the formation of ROCK/PDK1 complex and the increase in PDK1 activity and thereby sustains a vicious circle that contributes to prion disease progression. The inhibition of ROCK with Y-27632 or dimethylfasudil (i) rescues F-actin plasticity necessary for neuritogenesis, synapse connectivity and the integrity of axon/dendrites, and (ii) disrupts the ROCK/PDK1 complex, which lowers PDK1 activity and allows TACE to target back to the plasma membrane. Relocated TACE upon ROCK inhibition recovers its cleavage activity towards PrP^C^ and attenuates PrP^C^ conversion into PrP^Sc^. ROCK emerges as potential therapeutic target to combat prion diseases.

Our findings indicate that PrP^Sc^-induced ROCK overactivity contributes to neuronal cell demise at two levels: through (i) alterations of neuronal polarity, connectivity and neurotransmitter-associated functions and (ii) amplification of the production of neurotoxic PrP^Sc^. ROCK inhibition not only restores neuritogenesis and neurotransmitter-associated functions, but also reduces the production of PrP^Sc^, which thereby lengthens the survival of prion-infected mice (Fig 7).

The rise in ROCK activity in prion-infected cells originates from loss of PrP^C^ regulatory function towards the RhoA-ROCK-LIMK-cofilin pathway [15] upon PrP^C^ conversion into PrP^Sc^. Downstream modifications of F-actin structure and dynamics impair the onset of neurites by differentiating 1C11 neuronal stem cells, disrupt synapse connectivity and provoke neurite degradation in primary neuronal cultures. Our data provide evidence that PrP^Sc^-induced ROCK overactivation exerts a dominant negative effect on neuronal polarity since the sole inhibition of ROCK is sufficient to restore neurite sprouting and protect mature neurons from prion-induced axon and dendrite alterations. The beneficial effect afforded by ROCK inhibition on neuritogenesis and neuronal architecture relates to rescued cofilin-mediated severing of F-actin and improved actin dynamics in prion-infected cells. This however does not exclude that modulation of other signaling effectors known to regulate neuronal outgrowth downstream from ROCK, such as profilin IIa [45], also partakes to restoration of neuronal morphology upon ROCK inhibition.

Our data further show that prion infection of 1C11 neuronal stem cells alters the coupling between cell morphogenesis and the acquisition of neuronal functions. Inhibition of ROCK not only restores neuritogenesis but also rescues the overall neurotransmitter-associated functions. How the inhibition of ROCK permits to restore neuronal functions has however not been deciphered. Neuritogenesis and the onset of neurotransmitter-functions are interconnected events since the actin cytoskeleton directs the transport of specific mRNAs and proteins [46–48] and acts as a scaffold that orchestrates local protein translation [47,49,50] within axons and dendrites. Of note, all the mRNAs coding for neuronal functions are expressed in 1C11 cells at the stem cell stage but are dormant [51]. Prion infection does not impact on the expression level of these mRNAs (S4 Fig). The drastic reduction of Tryptophan Hydroxylase-2 (TPH2) expression measured in prion-infected 1C11^5-HT^ neurons might thus originate from abnormal trafficking of TPH2 mRNA and/or compromised translation. Interestingly, overphosphorylation of the translation initiation factor eiF2α, subsequent decrease of its activity and repression of protein synthesis have been shown to contribute to the progression of prion diseases [52]. Whether rescue of TPH2 protein synthesis upon ROCK inhibition relates to a restoration of eiF2α activity needs further investigation. By contrast with TPH2, the loss of Serotonin Transporter (SERT) functionality within an infectious context does not originate from reduced SERT expression, indicating that PrP^Sc^ interferes with the translation of specific mRNAs. Rescue of SERT activity upon ROCK inhibition may alternatively be linked to reduction of the intracellular concentration of serotonin-oxidized neurotoxins that are assumed to poison the SERT protein [23], corroborating the antioxidant effect of ROCK inhibition [53,54].

A major output of this work is the reduction, upon ROCK inhibition, of PrP^Sc^ level in prion-infected cells or in the brain of 22L-infected mice. Such reduction in brain PrP^Sc^ likely accounts for improved motor function and increased survival of prion-infected animals treated with the ROCK inhibitor. In this work, we decipher how ROCK contributes to the conversion of PrP^C^ into PrP^Sc^. Under physiological conditions ROCK-I acts as positive regulator of PDK1 activity. ROCK-I interacts with and phosphorylates PDK1. At a mechanistic level, prior autophosphorylation of Ser241 in the kinase domain of PDK1 is necessary to ROCK-I interaction and subsequent PDK1 phosphorylation by ROCK-I, suggesting that PDK1 phosphorylation at Ser241 induces conformational changes unmasking binding site(s) for ROCK-I and/or phosphorylatable residues by ROCK-I. Additional phosphorylation of PDK1 by ROCK-I improves the stability of the ROCK-I/PDK1 complex. In prion-infected cells, the rise in ROCK activity increases the pool of PDK1 molecules interacting with and phosphorylated by ROCK-I at the root of the PDK1 activity overboost within an infectious context (Fig 7). Such ROCK-dependent overstimulation of PDK1 activity in turn cancels plasma membrane TACE neuroprotective α-cleavage of PrP^C^ and thereby favors the production of PrP^Sc^ [24]. We previously showed that a rise in Src kinases-PI3K signaling in prion-infected cells also enhanced PDK1 activity [24], likely as the result of an accelerated PDK1 docking to the plasma membrane by phosphatidylinositol 3,4,5-trisphosphate and subsequent phosphorylation by Src kinases [41,43]. Inhibition of ROCK in prion-infected cells is however sufficient to decrease PDK1 activity up to its basal level as does the sole inhibition of Src kinases or PI3K [24], thus suggesting that the Src kinases-PI3K and ROCK pathways are both required for overstimulation of PDK1 activity within an infectious context.

Inhibition of ROCK has been shown to exert beneficial effects towards other amyloid-based neurodegenerative diseases such as Alzheimer’s (AD) [55] or Parkinson’s diseases [56,57]. In mouse models with AD-like pathology, decreased levels of neurotoxic amyloid Aβ peptides upon ROCK inhibition have been attributed to disruption of amyloidogenic processing of the amyloid precursor protein APP by the β-secretase BACE [58]. In brain samples from AD subjects as well as in three mouse models with AD-like pathology, PDK1 overactivity was also shown to contribute to the accumulation of Aβ40/42 peptides and the progression of AD by cancelling the non-amyloidogenic processing of APP by TACE [24]. By showing that ROCK overactivation is a novel pathogenic event in TSEs and that ROCK control PDK1 activity, we propose that the beneficial effect afforded by ROCK inhibition against AD also reflects down-regulation of PDK1 activity and rescue of TACE α-secretase activity towards APP. In any case, in the absence of effective medicine, further understanding the functional interplay between ROCK and PDK1 may help to design novel therapeutic strategies for amyloid-based neurodegenerative disorders.

## Materials and Methods

### Antibodies

The rat monoclonal CD9 antibody was a kind gift from E. Rubinstein (Inserm, Villejuif, France) [59]. The mouse monoclonal SAF32 PrP antibody was from SPI-Bio (Montigny Le Bretoneux, France). The mouse monoclonal ICSM33 PrP antibody was from D-Gen Limited (London, UK). The mouse monoclonal anti-CD29 (β1 integrin) antibody was from BD Transduction Laboratories (Lexington, KY, USA). The rat monoclonal 9EG7 anti-activated β1 integrin antibody [60] was from Pharmingen (San Diego, CA, USA). The mouse monoclonal antibody to α-tubulin was from Novus Biologicals (Littleton, CO, USA). The rabbit polyclonal antibody targeting phospho-LIMK1/2 (pThr505 and pThr508) was from AbCam (Cambridge, MA, USA). Rabbit polyclonal antibodies toward LIMK1 and cofilin were from Cell Signaling (Beverly, MA, USA). The rabbit polyclonal anti-phospho-Ser3-cofilin antibody was from Santa Cruz Biotechnology (SantaCruz, CA, USA). The rabbit polyclonal anti-TPH2 antibody was from Genway (San Diego, CA, USA). The rabbit polyclonal anti-SERT antibody was from Chemicon (Temecula, CA, USA). The rabbit polyclonal antibody to MAP2, the mouse polyclonal antibody to phospho-NFL200 (SMI31) and the guinea pig polyclonal anti-v-GLUT1 antibody were from EMD Millipore (Darmstadt, Germany). Rabbit polyclonal antibody to TACE was purchased from QED Bioscience (San Diego, CA, USA). The rabbit polyclonal antibodies to caveolin-1 (Cav-1) and focal adhesion kinase (FAK) were from Transduction laboratories (Lexington, KY, USA) and Santa Cruz Biotechnology (SantaCruz, CA, USA), respectively. The rabbit monoclonal ROCK-I and polyclonal ROCK-II and PDK1 antibodies were from Cell Signaling (Beverly, MA, USA). When nonspecified, primary antibodies were used at 0.5 μg ml^−1^ for western blot experiments and at 5 μg ml^−1^ for immunofluorescence experiments.

### Ethics statement

Adult C57Bl/6J mice were bred and underwent experiments in level-3 biological risk containment, respecting European guidelines for the care and ethical use of laboratory animals (Directive 2010/63/EU of the European Parliament and of the Council of 22 September 2010 on the protection of animals used for scientific purposes). Mice received intracerebral inoculation of the cerebellotropic 22L scrapie strain [44] (CNRS Strasbourg, France). All animal procedures were approved by the Comité Régional d’Ethique en Matière d’Expérimentation Animale de Strasbourg (France; CEEA35 ref AL/01/01/01/13) and the Animal Care and Use Committee at Basel University (Switzerland).

### Chronic intraperitoneal injection of Y-27632 or dimethylfasudil into mice

Mice were fasted overnight but allowed water *ad libitum* before the experiment. They were then anesthetized with isoflurane inhalation, and a midline incision was performed to insert into the peritoneum the polyethylene catheter of an osmotic pump (Alzet, Cupertino, CA, USA). Y-27632, dimethylfasudil or vehicle (1% DMSO in sterile normal saline buffer) was administered at a flow rate of 0.25 μL h^-1^, which corresponded to 100 μg per mouse per day (5 mg kg^−1^ per day for Y-27632 and 3 mg kg^−1^ per day for dimethylfasudil). Pumps were replaced every 4 weeks.

### Behavioral testing

Motor function in 22L-infected mice was assessed by the static rod test [61].

### Cell culture and prion infection

1C11 cells chronically infected or not by the mouse-adapted 22L or Fukuoka (Fk) strains [23] were grown and induced to differentiate along the serotonergic (1C11^5-HT^) pathway [22]. Primary CGNs were isolated from dissociated cerebella of 4- to 5-day-old C57Bl/6J mice and infected by the 22L strain [37,38].

### Cortico-striatal network with microfluidic chips

“Axon diode” microfluidic chips were designed and fabricated as previously described [34]. Briefly, Polydimethylsiloxane (Sylgard 184, PDMS, Dow Corning, Midland, MI, USA) was mixed with a curing agent (9:1 ratio) and degassed under vacuum. The resulting preparation was poured onto a polyester resin replicate and reticulated at 70°C for 2 h. The elastomeric polymer print was detached and two reservoirs were punched for each macro-channel. The resulting piece was cleaned with isopropanol and dried. The polymer print and a glass cover slip were treated for 200 sec in an air plasma generator (98% power, 0.6 mBar, Diener Electronic, Ebhausen, Germany) and bonded together. The chips were then coated with a solution of poly-D-lysine (10 μg ml^-1^, Sigma; St. Louis, MO, USA) overnight and washed with PBS before cell seeding.

Embryonic cortical and striatal neurons were obtained and cultured as previously described [34,35]. Briefly, cortices and ganglionic eminences (striatal neurons) were micro-dissected from E14 embryos of C57Bl/6J pregnant mice and digested with papaïn (20 U ml^-1^ in DMEM, Sigma Aldrich). Cortices and Striata were mechanically dissociated and neurons were collected by centrifugation. Cells were then re-suspended for plating in DMEM-Glutamax I (Life Technologies) supplemented with penicillin and streptomycin (Life Technologies), N2 and B27 supplements (Life Technologies) and 5% FBS (PAA). ~10^5^ cortical neurons and ~2.4 x 10^4^ striatal neurons were seeded respectively in cortical and striatal compartments, as described before [34]. Microfluidic chips were incubated at 37°C in humid atmosphere with 5% CO_2_. The culture medium was renewed every 7 days. Upon time of interest, cells were fixed using a 4% PFA, 4% sucrose solution diluted in PBS and further processed by immuno-cytochemistry with anti MAP2, α- tubulin and v-GLUT1 antibodies.

### Immunofluorescent labeling and F-actin visualization

Immunofluorescent labeling of PrP^C^, PrP^Sc^, β1 integrins, phospho-LIMK1/2, phospho-cofilin, TACE, MAP2, phospho-NFL200, and v-Glut1 was performed using standard protocols as reported in [15,37]. F-actin was stained using TRITC-phalloidin (Sigma-Aldrich, St. Louis, MO, USA) as in [15]. Labelings were analyzed using a Leica DMI6000 B microscope (Wetzlar, Germany) and subjected to image analysis with AQUA software [62].

### Cell extract preparation, PNGase assay and western blot analyses

Cells were washed in PBS/Ca^2+^/Mg^2+^ and incubated for 30 min at 4°C in lysis buffer (50 mM Tris-HCl pH 7.4, 150 mM NaCl, 5 mM EDTA, 1% Triton X-100, 1 mM Na_3_VO_4_ and protease inhibitors (Roche)). After centrifugation of the lysate (14,000*g*, 15 min), the protein concentration in the supernatant was measured with the bicinchoninic acid method (Pierce, Rockford, IL, USA). For the PNGase assay, protein extracts were incubated with 500 U N-glycosidase F (PNGase, New England Biolabs, Ipswich, MA, USA) for 1 h at room temperature. Solubilized proteins (20 μg) were resolved by 10% SDS-PAGE. After transfer, blocked membranes were incubated with SAF61 primary antibody. Bound antibodies were revealed by enhanced chemiluminescence detection (ECL, Amersham Pharmacia Biotech, Piscataway, NJ, USA). To standardize the results, membranes were rehybridized with an anti-α-tubulin antibody. The ratio between the truncated form of PrP (C1 fragment) and full-length PrP (Native) was evaluated by densitometric analyses using ImageQuant TL software (GE Healthcare, Little Chalfont, UK).

### Quantification of tryptophan hydroxylase activity

Determination of enzymatic functions was conducted on uninfected 1C11^5-HT^ cells, Fk-infected 1C11^5-HT^ cells and Rocki-Fk-1C11^5-HT^ cells. After two washings with cold PBS, cells were scraped and collected down by centrifugation (10,000*g*, 3 min, 4°C). Tryptophan hydroxylase (TPH) activity was measured radioenzymatically as in [23]. Briefly, cell extracts were incubated for 30 min at 37°C in an assay mixture containing 200 mM Na acetate, pH 6.1, 1 mM ferrous sulfate, 2 mM 6-methyl-H4-pterin, 40 mM 2-mercaptoethanol, 20 mM Na phosphate and 100 μM [^3^H]-L-tryptophan. TPH activity was determined by quantifying the production of [^3^H]_2_O in a liquid scintillation counter and expressed as pmol/30 min/mg of cell protein extract.

### Determination of 5-HT, 5-HIAA and 5-HT oxidized products

The contents in 5-HT and related metabolites were measured by HPLC combined to electrochemical (EC) detection as described in [23].

### Radioligand binding experiments

The presence of vesicular monoamine transporter (VMAT) sites was assessed through [^3^H]-tetrabenazine binding as in [23]. [^3^H]-paroxetine binding to functional 5-HT transporter (SERT) was carried out as in [33].

### Phospho-Ser3-cofilin level and PrP^Sc^ deposition in the brains of prion-infected mice

Prion-infected (*n* = 3) and control uninfected (*n* = 6) mice were anaesthetized with ketamine (125 mg kg^-1^) and xylazine (17 mg kg^-1^) and perfused via cardiac aorta with 4% paraformaldehyde at 130 days after infection. The cerebellum was removed and cryoprotected in sucrose 30% before freezing in isopentane at -80°C. Transverse cryostat sections (30 μm-thick) were cut and submitted floating to classical immuno-peroxidase staining of PrP^Sc^ and phosphorylated cofilin using SAF32 (1 μg ml^−1^) and anti-phospho-Ser3-cofilin (2 μg ml^−1^; Santa Cruz, CA, USA) antibodies, respectively. The specificity of PrP^Sc^ immunodetection was achieved by denaturing the PrP^C^ by incubation of the sections in proteinase K (10 μg ml^−1^) for 10 min at 37°C and subsequently in 3.4 M guanidine thiocyanate for 15 min. The PrP- and phospho-Ser3-cofilin-bound antibodies were visualized using biotinylated anti-mouse or anti-rabbit immunoglobulins (SouthernBiotech, Birmingham, AL, USA), respectively, and the Vectastain Elite kit (Vector Labs, Burlingame, CA, USA).

### ROCK inhibition

ROCK activity was inhibited with dimethylfasudil (Calbiochem, San Diego, CA, USA) or Y-27632 (Tocris Bioscience, Ellisville, MO, USA).

### PrP^res^ quantification

The amount of proteinase K–resistant PrP (PrP^res^) in infected cell lysates or brain extracts of 22L-infected mice infused or not with Y-27632 or dimethylfasudil were determined using a PrP-specific sandwich ELISA [24,63] after proteinase K digestion (10 μg ml^−1^) for 1 h at 37°C.

### Sucrose gradient fractionation of cell membranes

TACE was detected by western blot analysis after sucrose gradient membrane fractionation of cell or cerebellar extracts performed under detergent-free conditions to isolate low buoyant fractions enriched in caveolin-1 proteins [24,64].

### Measurement of PDK1 activity

PDK1 activity was measured in cell lysates or cerebellar extracts using a fluorescently labeled PDK1 substrate (5FAM-ARKRERTYSFGHHA-COOH, Caliper Life Sciences, Hanover, MD, USA) as reported in [24,64]. The relative amounts of substrate peptide and product phospho-peptide were determined using a Caliper EZ-reader (Caliper Life Sciences, Hanover, MD, USA).

### ROCK immunosequestration

ROCK-I or ROCK-II immunosequestration was performed by cell bombardment with tungsten microprojectiles coated with antibody to ROCK-I or ROCK-II [24,64].

### ROCK-I immunoprecipitation

ROCK-I immunoprecipitation was performed according to standard protocols by using protein A-Sepharose beads (Amersham Pharmacia Biotech, Picataway, NJ, USA) coupled to anti-ROCK-I antibody and 100 μg of cell lysates or cerebellar extracts. Immunoprecipitates were analyzed by western blotting using anti-ROCK-I and anti-PDK1 antibodies.

### Cell metabolic labeling with [^32^P]-orthophosphate

[^32^P]-orthophosphate labeling was performed as in [65]. Briefly, the cell culture medium was removed and cells were thoroughly washed with phosphate-free DMEM to eliminate any residual phosphate-containing medium. [^32^P]-orthophosphate (40.7 Gbq mmol^-1^, GE Healthcare, Little Chalfont, UK) was added to the cell culture at a final concentration of 18.5 Mbq ml^-1^. After 2 h, the labeling medium was removed and the cells were lyzed after extensive washing.

### Cell transfection of S241A PDK1 mutant

Endogenously expressed wild type PDK1 was constitutively repressed in the 1C11 cell system (PDK1^null^-cells) upon cell transfection of a shRNA of the *pdk1* gene [24] following the same procedure as in [15]. The mutated S241 PDK1 mutant was built by polymerase chain reaction and eight silent mutations were further introduced into the S241A *pdk1* gene sequence corresponding to the siRNA hybridization zone [15]. The mutated S241 *pdk1* gene was cloned into the pEBG-2T expression vector [66]. For expression of the S241A PDK1 mutant, PDK1^null^-cells were transfected with 10 μg of the pEBG-2T construct as in [67] and left to grown for 36 h. Reconstituted cells were then lyzed and assayed for PDK1 expression, PDK1 activity, PDK1 interaction with ROCK-I and PDK1 phosphorylation by ROCK-I after cell metabolic labeling with [^32^P]-orthophosphate.

### Data analysis

An analysis of variance of the cell/animal response group was performed using Kaleidagraph software (Synergy Software, Reading, PA, USA). Values are given as means ± s.e.m. Significant responses (*P* < 0.05) are marked by symbols (#,*,†,‡) and their corresponding *P* values are provided in figure legends. Survival times were analyzed by Kaplan-Meier survival analysis using a log-rank test for curve comparisons. When non-specified experiments were performed in three to five times in triplicates.

## Supporting Information

S1 Fig Inhibition of ROCK rescues neuritogenesis in prion-infected 1C11 cells. Phase pictures of control 1C11^5-HT^ neuronal cells at day 4 of the serotonergic program and Fk- or 22L-infected 1C11 cells induced to differentiate along the serotonergic pathway for 4 days in the absence or presence of two distinct ROCK inhibitors, Y-27632 (100 μM) or dimethylfasudil (2 μM). Scale bars, 50 μm.
(TIF)

S2 Fig ROCK overactivation in 22-infected CGNs promotes TACE internalization in a PDK1-dependent manner. (**A**) Immunofluorescent labeling of TACE at the surface of 22L-infected CGNs treated or not with the ROCK inhibitor Y-27632 (100 μM) for 1 h versus uninfected cells. Scale bar, 50 μm. (**B**) Western-blot analysis (top) of the C1 fragment of PrP (C1) and full-length PrP (Native) in 22L-infected CGNs treated or not with Y-27632 (100 μM) or a combination of Y-27632 (100 μM) and TAPI-2 (100 μM) for 6h *vs*. uninfected CGNs. Ratio (bottom) of C1/Native full-length PrP. (**C**) PDK1 activity in 22L-infected CGNs treated or not with Y-27632 compared to uninfected CGNs. Values are the mean ± s.e.m. * *P* < 0.01 versus non treated uninfected CGNs. ** *P* < 0.01 versus non treated 22L-infected CGNs. # *P* < 0.05 versus non treated uninfected CGNs. ## *P* < 0.05 versus non treated 22L-infected CGNs. ### *P* < 0.05 versus 22L-infected CGNs treated with Y-27632.
(TIF)

S3 Fig ROCK inhibition with dimethylfasudil attenuates prion disease in mice. (**A**) Survival curves of SHAM and 22L-inoculated mice via the intracerebellar route (i.c.b.) infused or not with the ROCK inhibitor dimethylfasudil by intraperitoneal injection (i.p.) starting at 130 days after infection (3 mg per kg body weight per day; 0.25 μl h^-1^). n = 10 mice per group. (**B**) Western blot and histogram quantifications for phosphorylated cofilin on Ser3 in 22L-infected mice infused or not with dimethylfasudil versus SHAM mice. n = 4 per condition. (**C**) Static rod test between 130 and 160 days after infection in 22L-infected mice treated with dimethylfasudil. n = 5 per group for mice treated with dimethylfasudil. n = 10 per group for untreated mice. (**D**) Left, Western-blot for proteinase K-resistant PrP^Sc^ in brain extracts from SHAM and 22L-infected mice infused or not with dimethylfasudil. Right, post-mortem quantification of proteinase K-resistant PrP^Sc^ in brains of 22L-infected mice treated or not with dimethylfasudil. n = 7 for each condition. (**E**) ROCK immunoprecipitation followed by PDK1 western blotting in cerebellar extracts of 22L-infected mice treated or not with dimethylfasudil versus SHAM mice. n = 6 for each condition. (**F**) PDK1 activity in cerebellar extracts of 22L-infected mice treated or not with dimethylfasudil versus SHAM mice. n = 6 for each condition. Values are the mean ± s.e.m. * *P* < 0.01 versus SHAM mice. ** *P* < 0.01 versus 22L-infected mice. # *P* < 0.05 versus SHAM mice. ## *P* < 0.05 versus 22L-infected mice.
(TIF)

S4 Fig Prion infection does not impact on TPH2 and SERT transcription in 1C11 precursor cells. RT-PCR analysis of TPH2 and SERT transcripts in 1C11 and Fk-infected 1C11 cells was performed as described in [47]. GAPDH was used for normalization.
(TIF)

S1 Table Inhibition of ROCK with Y-27632 (100 μM) or dimethylfasudil (2 μM) restores neuritogenesis in Fk-1C11 and 22L-1C11 cells exposed for 4 days to serotonergic inducers. (TIF)
