# Supplementary figures and images for: Double-Edge Sword of Sustained ROCK Activation in Prion Diseases through Neuritogenesis Defects and Prion Accumulation
Source: PLoS Pathog. 2015 Aug 4;11(8):e1005073. doi: 10.1371/journal.ppat.1005073 (PMC4524729; doi:10.1371/journal.ppat.1005073)

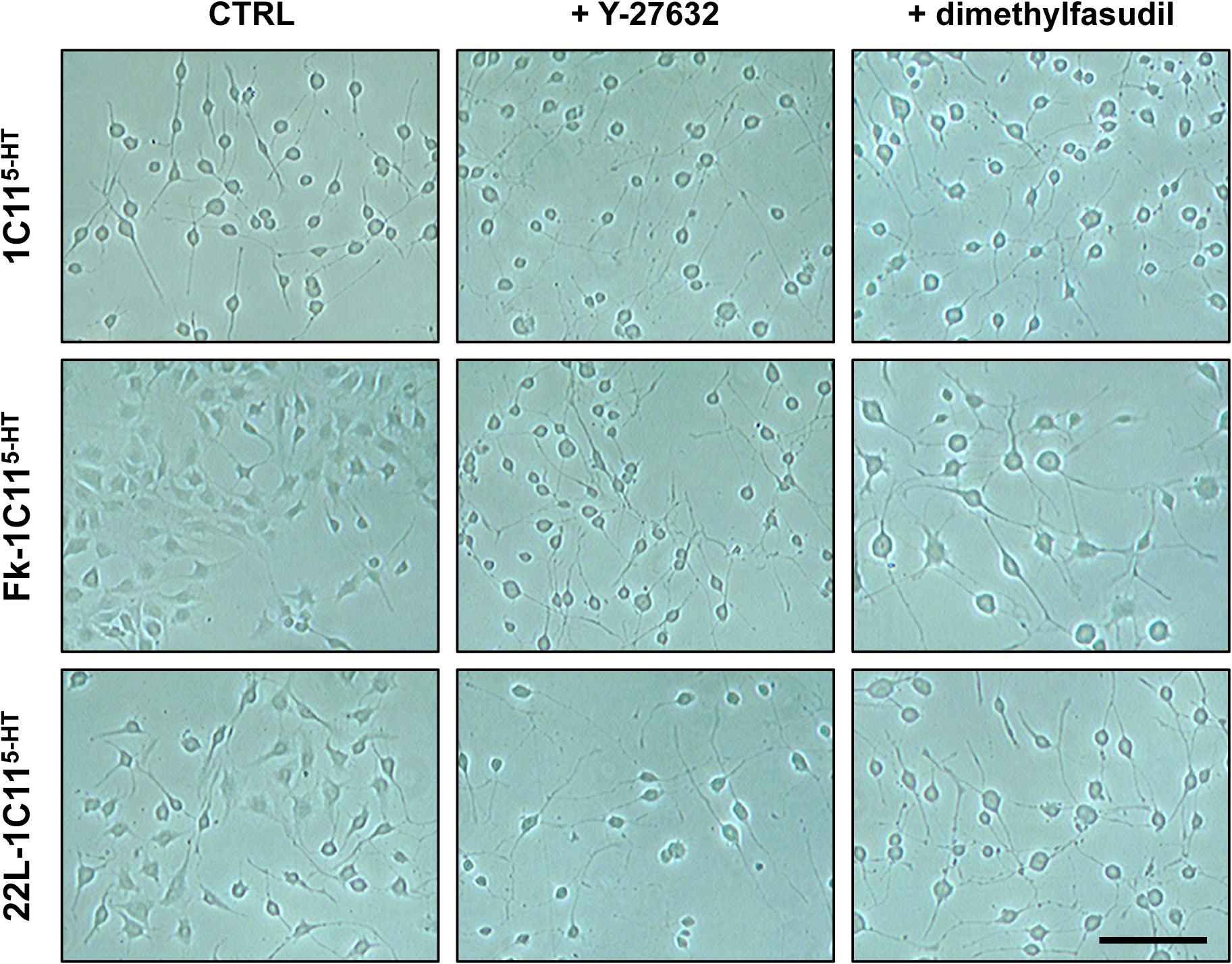

Supplement: S1 Fig — Phase pictures of control 1C115-HT neuronal cells at day 4 of the serotonergic program and Fk- or 22L-infected 1C11 cells induced to differentiate along the serotonergic pathway for 4 days in the absence or presence of two distinct ROCK inhibitors, Y-27632 (100 μM) or dimethylfasudil (2 μM). Scale bars, 50 μm. (TIF) [file ppat.1005073.s001.tif]

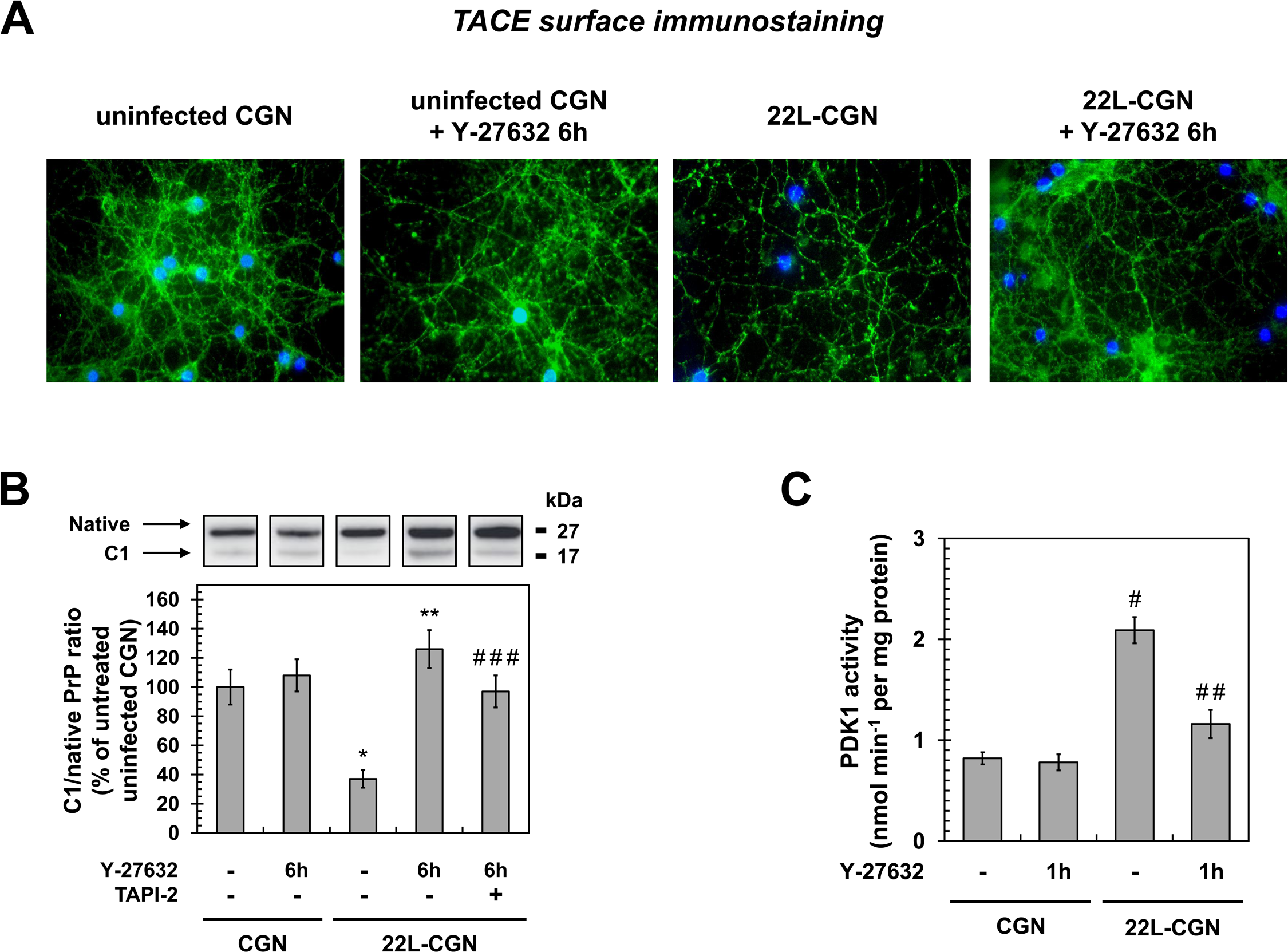

Supplement: S2 Fig — (A) Immunofluorescent labeling of TACE at the surface of 22L-infected CGNs treated or not with the ROCK inhibitor Y-27632 (100 μM) for 1 h versus uninfected cells. Scale bar, 50 μm. (B) Western-blot analysis (top) of the C1 fragment of PrP (C1) and full-length PrP (Native) in 22L-infected CGNs treated or not with Y-27632 (100 μM) or a combination of Y-27632 (100 μM) and TAPI-2 (100 μM) for 6h vs. uninfected CGNs. Ratio (bottom) of C1/Native full-length PrP. (C) PDK1 activity in 22L-infected CGNs treated or not with Y-27632 compared to uninfected CGNs. Values are the mean ± s.e.m. * P < 0.01 versus non treated uninfected CGNs. ** P < 0.01 versus non treated 22L-infected CGNs. # P < 0.05 versus non treated uninfected CGNs. ## P < 0.05 versus non treated 22L-infected CGNs. ### P < 0.05 versus 22L-infected CGNs treated with Y-27632. (TIF) [file ppat.1005073.s002.tif]

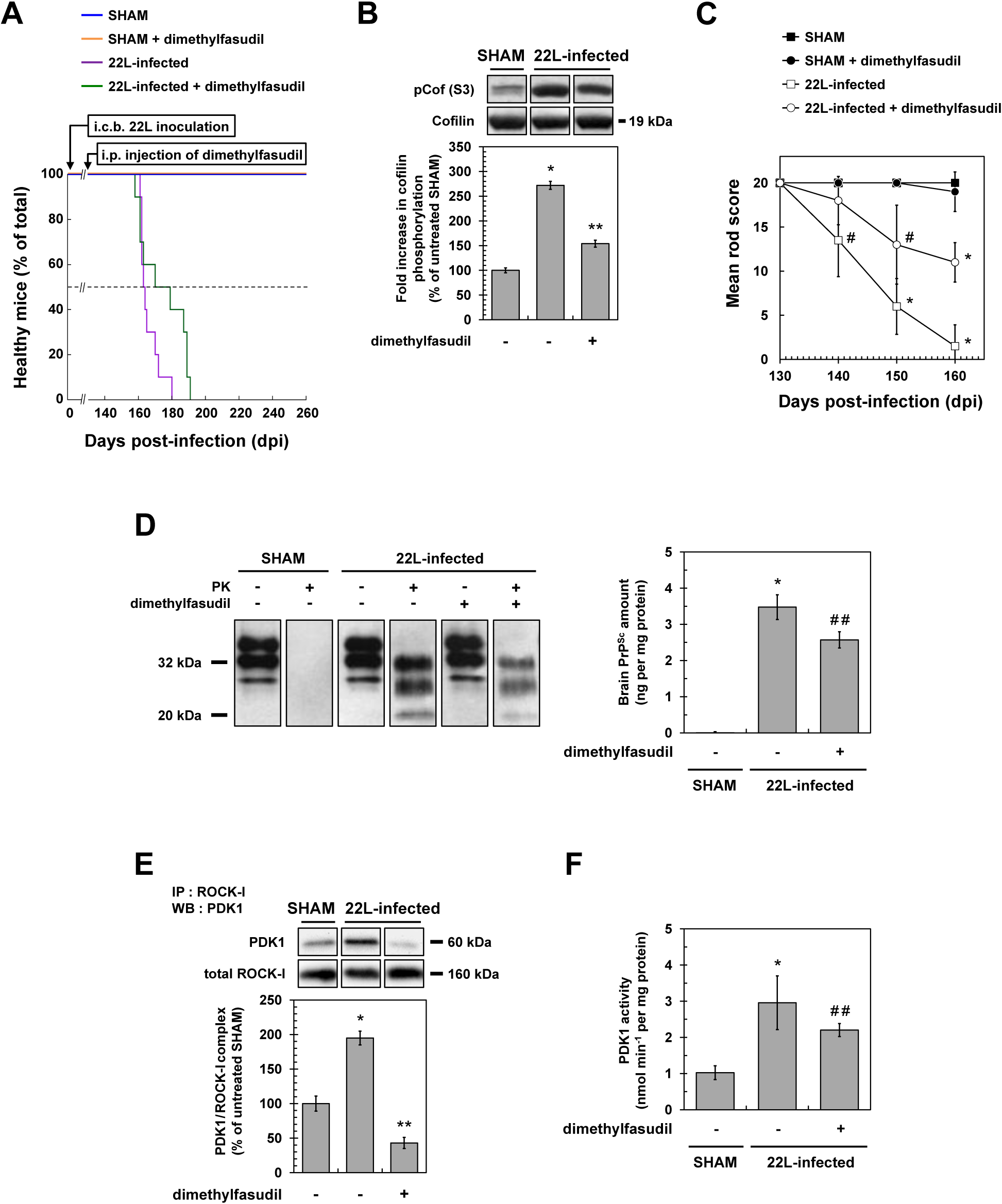

Supplement: S3 Fig — (A) Survival curves of SHAM and 22L-inoculated mice via the intracerebellar route (i.c.b.) infused or not with the ROCK inhibitor dimethylfasudil by intraperitoneal injection (i.p.) starting at 130 days after infection (3 mg per kg body weight per day; 0.25 μl h-1). n = 10 mice per group. (B) Western blot and histogram quantifications for phosphorylated cofilin on Ser3 in 22L-infected mice infused or not with dimethylfasudil versus SHAM mice. n = 4 per condition. (C) Static rod test between 130 and 160 days after infection in 22L-infected mice treated with dimethylfasudil. n = 5 per group for mice treated with dimethylfasudil. n = 10 per group for untreated mice. (D) Left, Western-blot for proteinase K-resistant PrPSc in brain extracts from SHAM and 22L-infected mice infused or not with dimethylfasudil. Right, post-mortem quantification of proteinase K-resistant PrPSc in brains of 22L-infected mice treated or not with dimethylfasudil. n = 7 for each condition. (E) ROCK immunoprecipitation followed by PDK1 western blotting in cerebellar extracts of 22L-infected mice treated or not with dimethylfasudil versus SHAM mice. n = 6 for each condition. (F) PDK1 activity in cerebellar extracts of 22L-infected mice treated or not with dimethylfasudil versus SHAM mice. n = 6 for each condition. Values are the mean ± s.e.m. * P < 0.01 versus SHAM mice. ** P < 0.01 versus 22L-infected mice. # P < 0.05 versus SHAM mice. ## P < 0.05 versus 22L-infected mice. (TIF) [file ppat.1005073.s003.tif]

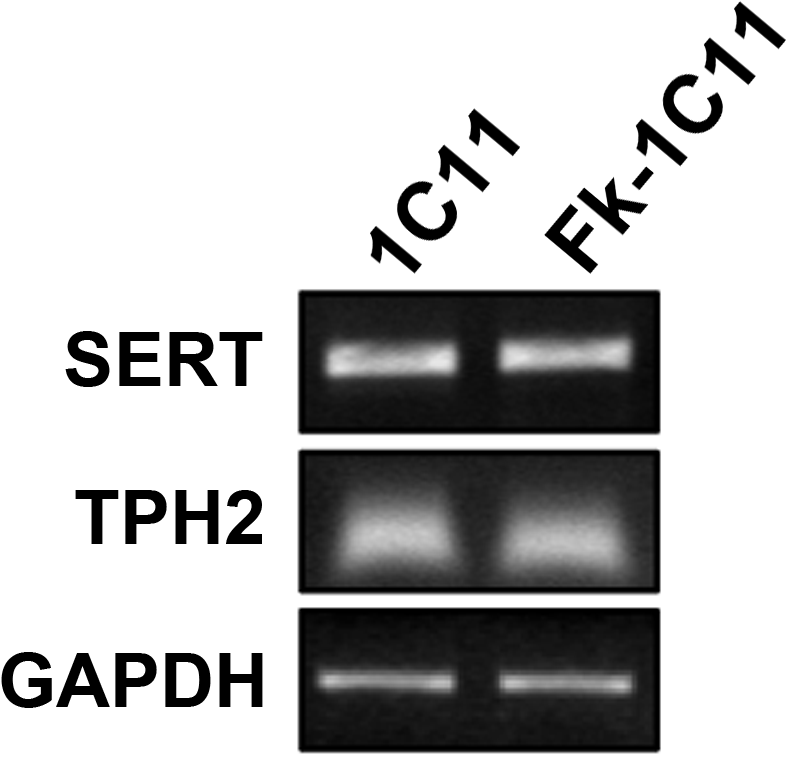

Supplement: S4 Fig — RT-PCR analysis of TPH2 and SERT transcripts in 1C11 and Fk-infected 1C11 cells was performed as described in [47]. GAPDH was used for normalization. (TIF) [file ppat.1005073.s004.tif]

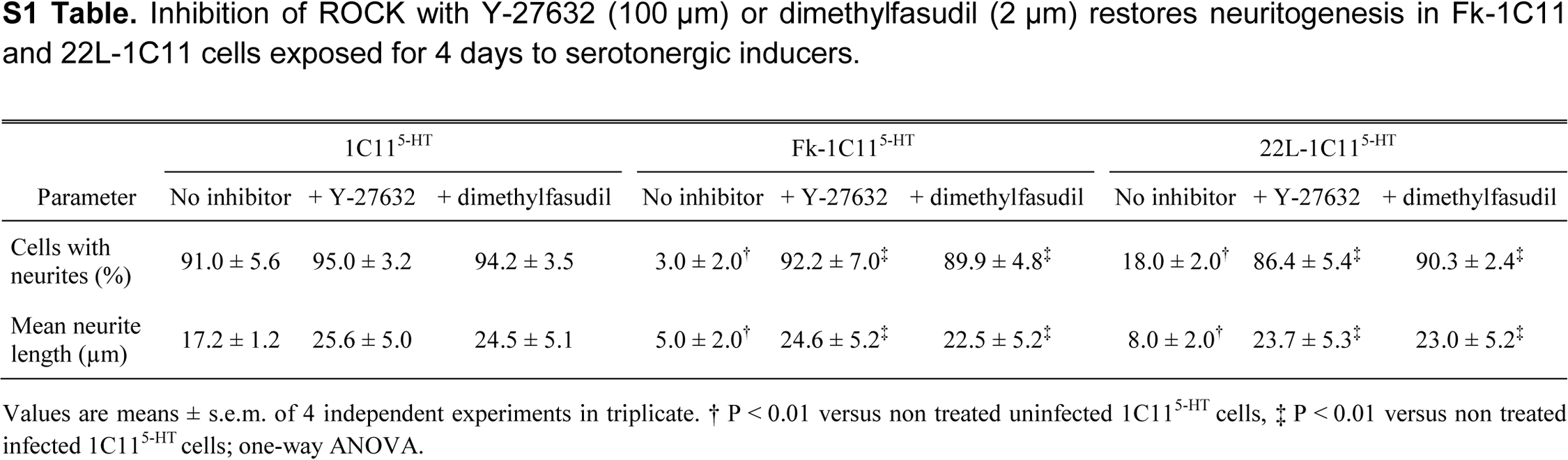

Supplement: S1 Table — (TIF) [file ppat.1005073.s005.tif]
